# IL-1B drives opposing responses in primary tumours and bone metastases; harnessing combination therapies to improve outcome in breast cancer

**DOI:** 10.1038/s41523-021-00305-w

**Published:** 2021-07-21

**Authors:** Claudia Tulotta, Diane V. Lefley, Charlotte K. Moore, Ana E. Amariutei, Amy R. Spicer-Hadlington, Lewis A. Quayle, Russell O. Hughes, Khawla Ahmed, Victoria Cookson, Catherine A. Evans, Jayakumar Vadakekolathu, Paul Heath, Sheila Francis, Emmanuel Pinteaux, A. Graham Pockley, Penelope D. Ottewell

**Affiliations:** 1 Department of Oncology and Metabolism, Weston Park Cancer Centre, University of Sheffield, Sheffield, UK; 2 John van Geest Cancer Research Centre, School of Science and Technology, Nottingham Trent University, Nottingham, UK; 3 Sheffield Institute for Translational Neuroscience, University of Sheffield, Sheffield, UK; 4 Department of Infection, Immunity and Cardiovascular Disease, University of Sheffield, Sheffield, UK; 5 Faculty of Biology, Medicine and Health, University of Manchester, Manchester, UK

**Keywords:** Breast cancer, Tumour immunology

## Abstract

Breast cancer bone metastasis is currently incurable, ~75% of patients with late-stage breast cancer develop disease recurrence in bone and available treatments are only palliative. We have previously shown that production of the pro-inflammatory cytokine interleukin-1B (IL-1B) by breast cancer cells drives bone metastasis in patients and in preclinical in vivo models. In the current study, we have investigated how IL-1B from tumour cells and the microenvironment interact to affect primary tumour growth and bone metastasis through regulation of the immune system, and whether targeting IL-1 driven changes to the immune response improves standard of care therapy for breast cancer bone metastasis. Using syngeneic IL-1B/IL1R1 knock out mouse models in combination with genetic manipulation of tumour cells to overexpress IL-1B/IL1R1, we found that IL-1B signalling elicited an opposite response in primary tumours compared with bone metastases. In primary tumours, IL-1B inhibited growth, by impairing the infiltration of innate immune cell subsets with potential anti-cancer functions but promoted enhanced tumour cell migration. In bone, IL-1B stimulated the development of osteolytic metastases. In syngeneic models of breast cancer, combining standard of care treatments (Doxorubicin and Zoledronic acid) with the IL-1 receptor antagonist Anakinra inhibited both primary tumour growth and metastasis. Anakinra had opposite effects on the immune response compared to standard of care treatment, and its anti-inflammatory signature was maintained in the combination therapy. These data suggest that targeting IL-1B signalling may provide a useful therapeutic approach to inhibit bone metastasis and improve efficacy of current treatments for breast cancer patients.

## Introduction

Breast cancer (BC) is the second most common cancer worldwide (2.26 million cases in 2020), accounting for 685,000 deaths (https://www.who.int/news-room/fact-sheets/detail/cancer, 2020). The majority of cancer-related deaths result from tumours spreading to distal sites after which point the disease is currently incurable.

Bone is the most common metastatic site in BC—75% of patients with late-stage BC experience recurrence in bone—followed by lung, liver and brain^1–3^. Once tumour cells disseminate in bone they enter state of dormancy before cell-autonomous mechanisms and cellular interactions with the bone microenvironment stimulates proliferation and development of overt metastases^4–7^. The role of the immune system in controlling metastatic tumour cell dormancy in bone remains to be explored^8^. As current treatments for metastatic breast cancer are predominantly palliative, there is an urgent clinical need to identify new biomarkers of metastatic BC and to develop more effective therapies^9^.

We recently identified the pro-inflammatory cytokine Interleukin-1B (IL-1B) as a marker of breast cancer bone metastasis and showed that pharmacological targeting of IL-1B or its receptor (IL1R1) impairs bone metastasis^10–13^. We and others have previously shown that tumour-derived IL-1B drives EMT^11,14^. We suggested that inhibition of IL-1B using Canakinumab supports an epithelial rather than a mesenchymal phenotype thus, favoring tumour proliferation while inhibiting EMT and therefore metastasis^11^.

The tumour cell is not the only source of IL-1B; cells in the microenvironment, including immune cells, endothelial cells, osteoblasts and bone marrow cells produce IL-1B. The arrival of tumour cells in bone triggers further production of IL-1B from tumour cells as well as IL-1B release from cells within the bone marrow^11^. This microenvironmental increase in IL-1B promotes metastatic colonisation of bone via Wnt signalling^15^ as well as stimulating expansion of the bone metastatic (osteoblastic and vascular) niches^11^. Moreover, IL-1B produced by macrophages infiltrating the primary tumour, initiates a systemic neutrophilic inflammatory response that ultimately results in CD8^+^ T cell inhibition, immunosuppression and metastasis to the lungs^16,17^.

Since IL-1B causes immunosuppression^18^, inhibition of this cytokine in combination with immunotherapies has been found to promote anti-tumour immunity and impair primary tumour growth^19^. Therefore, administration of anti-IL-1 therapies in combination with immune-stimulatory drugs may provide effective therapeutic options for patients with breast cancer. The chemotherapeutic compound Doxorubicin is an anthracycline commonly used to treat breast cancer and bone metastasis. This drug stops tumour proliferation by targeting the topoisomerase 2 enzyme and induces inflammatory cell death (ICD)^20,21^ leading to infiltration of immune cells.

In addition to chemotherapy, breast cancer bone metastases are commonly treated with zoledronic acid. We have previously shown that combining doxorubicin with zoledronic acid synergistically increases the anti-tumour effects of doxorubicin when given in sequence^22–24^. When translated into the clinic, adding zoledronic acid to standard of care in an adjuvant setting reduced bone metastases in all patient groups. However, this combination only had life prolonging benefits in women with established menopause by reducing tumour recurrence in any tissue. In pre-menopausal women, the same treatment resulted in increased development of soft tissue metastasis^25^. Interestingly, patients who did worse with this combination treatment had higher levels of IL-1B in their primary tumours than those that responded well^11^. It is therefore possible that adding anti-IL-1 treatment to doxorubicin and zoledronic acid may increase the anti-metastatic effects of these drugs in a pre-menopausal setting.

In the current study, we investigate how IL-1B from different environments influences immune cell abundance, driving primary tumour growth and bone metastasis and whether this has an effect on the response to standard of care therapies. We hypothesise that combining doxorubicin and zoledronic acid with anti-IL-1 treatments may increase the therapeutic effects of standard of care by increasing tumour cell killing at both primary and metastatic sites. We found that both tumour and microenvironment-derived IL-1B drive the infiltration of immune cells which may harbour anti-tumour functions in breast primary tumours, however, in bone, IL-1B supports metastasis formation. By maintaining its anti-inflammatory effect, anti-IL-1 therapy leads to increased efficacy of standard of care treatment for bone metastasis. Our new data strongly suggest that adding anti-IL-1 treatment to standard of care therapy is a potential effective treatment for patients with breast cancer.

## Results

### Microenvironment-derived IL-1B controls primary breast tumour development

**Fig. 1:**
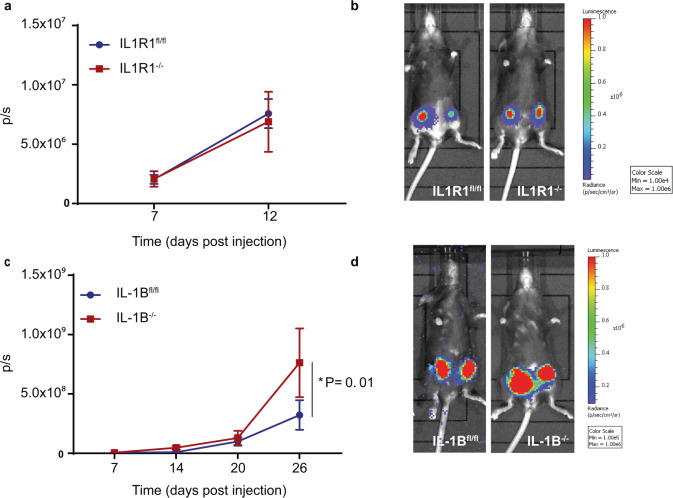
Depletion of IL-1B but not IL1R1 from the microenvironment promotes primary tumour growth. **a** Quantification (photons/sec (p/s)) of primary tumour growth in IL1R1^fl/fl^ (*n* = 8 primary tumours from *n* = 4 mice) and IL1R1^−/−^ (*n* = 8 primary tumours from *n* = 4 mice) mice up to 12 days of post-orthotopic injection of E0771-luc2-V5-GFP cells and **b** correspondent micrographs. **c** Quantification (p/s) of primary tumour growth in IL-1B^fl/fl^ (*n* = 7) and IL-1B^−/−^ (*n* = 6) mice up to 26 days of post-orthotopic injection of E0771-luc2- GFP cells and **d** correspondent micrographs. IL-1B^fl/fl^: *n* = 13 primary tumours from *n* = 7 mice (day 7 and 14); *n* = 12 primary tumours from *n* = 6 mice (day 20 and 26). IL-1B^−/−^: *n* = 12 primary tumours from *n* = 6 mice (day 7); *n* = 10 primary tumours from *n* = 6 mice (day 14, 20, and 26). 2.3-fold increase in primary tumour growth in IL-1B^−/−^ mice (7.6 × 10^8^ p/s) compared to IL-1B^fl/fl^ mice (3.2 × 10^8^ p/s) (*P* = 0.01). Data are mean +/− SEM, Two-way ANOVA with Sidak’s post-hoc test.

We previously showed that pharmacological inhibition of IL-1B signalling using the IL-1Ra, Anakinra, did not affect the growth of the primary tumour, whereas treatment with an anti-IL-1B antibody, Canakinumab, increased proliferation. Since pharmacological treatments can equally affect the tumour as well as the stroma and previous experiments had used immune-compromised mice, we next addressed the contribution of microenvironment-derived IL-1B on primary tumour growth and immune response to breast cancer in orthotopic models. To model a microenvironment in which IL1R1 or IL-1B is absent, mice with a ubiquitous IL1R1 KO (K14Cre; Il1r1^fl/fl^)^26,27^ or IL-1B KO (PGKCre; Il1b^fl/fl^) (Supplementary Fig. 1) were used along with correspondent IL1R1^fl/fl^ or IL-1B^fl/fl^ mice as control. In these models, we found that inhibition of IL1R1 did not affect primary tumour growth (Fig. 1a, b), whereas inhibition of IL-1B resulted in increased primary tumour growth (Fig. 1c, d), therefore recapitulating the effect that we had previously seen following pharmacological inhibition of IL-1B signalling^11^.

### Microenvironment-derived IL-1B impairs primary tumour growth whilst recruiting different subsets of innate immune cells

**Fig. 2:**
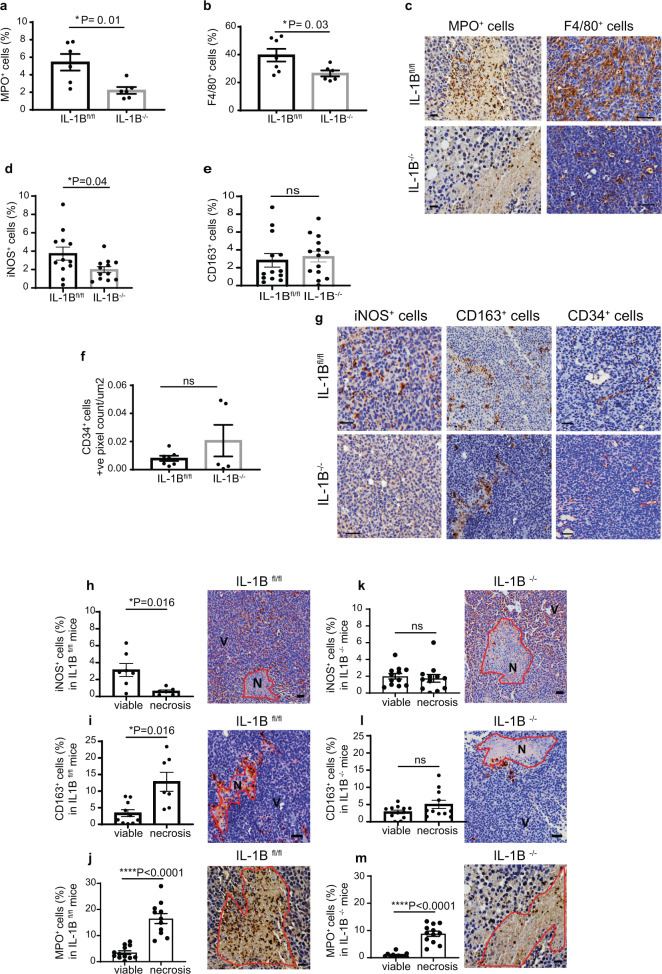
Microenvironment-derived IL-1B drives the infiltration of innate immune cells with putative anti-tumour function and promotes immune cell subset positioning in primary breast tumours. **a** MPO^+^ neutrophils (*P = 0.01) and **b** F4/80^+^ macrophages (*P = 0.03) in the primary tumour of IL-1B^fl/fl^ and IL-1B^−/−^ mice. Each dot represents a tissue section from a primary tumour isolated from each mouse. **c** Representative IHC micrographs of MPO and F4/80 staining. **d** iNOS^+^ (*P = 0.04), **e** CD163^+^ (ns) and **f** CD34^+^ (ns) cells in the primary tumour of IL-1B^fl/fl^ and IL-1B^−/−^ mice. **d**, **e** Each dot represents an inner or outer section from a primary tumour obtained from each mouse. **f** Each dot represents a tissue section from a primary tumour isolated from each mouse. **g** Representative IHC micrographs of iNOS, CD163 and CD34 staining. Data are mean +/− SEM Two-tailed unpaired *t*-test with Welch’s correction. Scale bar: 50 µm. **h** iNOS^+^ cells in viable and necrotic areas of the primary tumour (tissue section from the tumour core) (*P* = 0.016) of IL-1B^fl/fl^ mice determined by IHC. **i** CD163^+^ cells in viable and necrotic areas of the primary tumour (*P* = 0.016) of IL-1B^fl/fl^ mice determined by IHC. **j** MPO^+^ neutrophils in viable and necrotic areas of IL-1B^fl/fl^ (*P* < 0.0001). **k** iNOS^+^ cells in viable and necrotic regions of primary tumours from IL-1B^−/−^ mice. **l** CD163^+^ cells in viable and necrotic areas of primary tumours from IL-1B^−/−^ mice. **m** MPO^+^ neutrophils in viable and necrotic areas of IL-1B^−/−^ (*P* < 0.0001) mice. Data are shown as mean +/− SEM, Two-tailed unpaired *t*-test with Welch’s correction, ns non-significant. Scale bar: 50 µm in **h**, **i**, **k**, **l**. Scale bar: 20 µm in **j** and **m**.

As genetic removal of IL-1B or IL1R1 in syngeneic mouse models or pharmacological suppression of this cytokine/receptor in immune-compromised (T and B cell deficient) mouse strains led to the same effects, we hypothesised that IL-1B controls the growth of the primary tumour independently of the adaptive immune response. We therefore investigated the contribution of IL-1 driven changes in primary tumour growth focusing on infiltration of innate immune cell subsets using immunohistochemistry (IHC). Our data showed that lack of IL-1B resulted in increased primary tumour growth and decreased infiltration of MPO^+^ neutrophils and F4/80^+^ macrophages (Fig. 2a–c). Next, we evaluated the infiltration of specific subsets of iNOS^+^ anti-tumour immune cells (M1-like macrophages)^28^ and CD163^+^ pro-tumour M2-like macrophages^29^. Since M2-like macrophages have pro-angiogenic functions^30^, we stained for CD34^+^ blood vessels within the primary tumour. We found that IL-1B regulates iNOS^+^ immune cell infiltration, without affecting either the accumulation of CD163^+^ immune cells or the growth of tumour-associated CD34^+^ blood vessels (Fig. 2d–g). Furthermore, we observed that iNOS^+^ immune cells localise specifically in viable regions, whereas CD163^+^ M2-like macrophages and MPO^+^ neutrophils were found in areas of necrosis (Fig. 2h–j). IL-1B ablation in the microenvironment removed this spatial bias, resulting in an equal number of iNOS^+^ and CD163^+^ macrophages in both areas of tumour viability and necrosis (Fig. 2k, l). MPO^+^ neutrophils localised in areas of necrosis and their distribution was not altered upon IL-1B ablation in the microenvironment (Fig. 2m). Taken together, these findings suggest that microenvironment-derived IL-1B may control breast primary tumour growth by driving an innate immune response, with potential anti-tumour functions.

### Tumour-derived IL-1B limits primary tumour development and re-establishes the infiltration of immune cells with potential anti-tumour functions

**Fig. 3:**
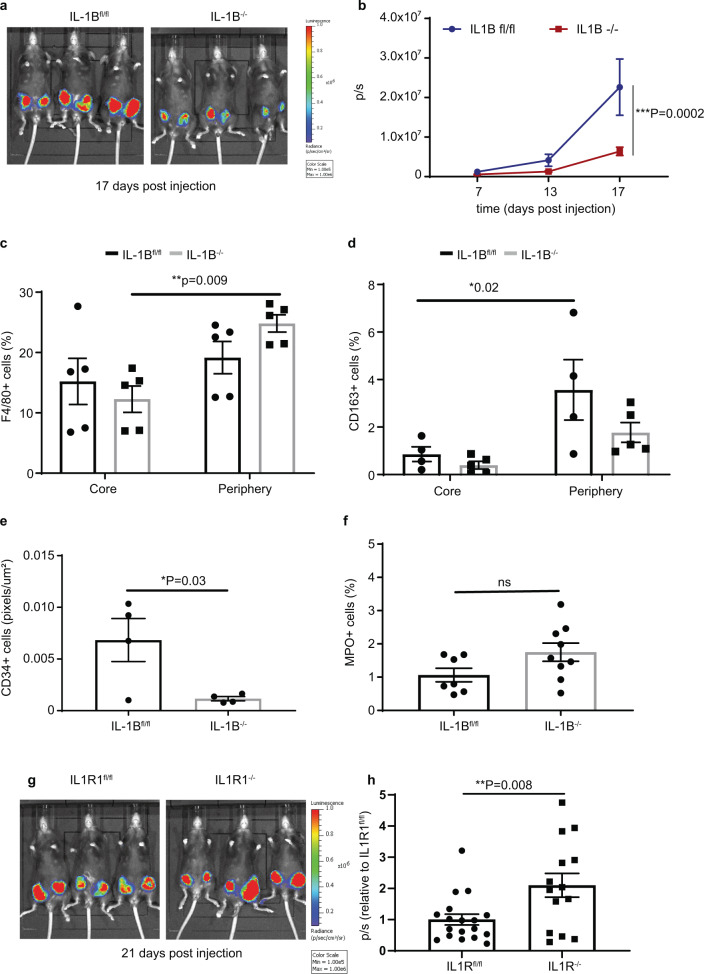
Tumour-derived IL-1B restores the infiltration of innate immune cells that may display anti-tumour functions and inhibits primary tumour growth in an IL-1B deficient microenvironment. **a**, **b** Images and quantification of primary tumour development in IL-1B^fl/fl^ (*n* = 8 primary tumours from *n* = 4 mice) and IL-1B^−/−^ (*n* = 10 primary tumours from *n* = 5 mice) mice after intra-ductal administration of E0771 Luc2 V5 IL-1B-GFP cells. Data are mean +/− SEM, Two-way ANOVA with Sidak’s post-hoc test. **c**, **d** Quantification of F4/80^+^ macrophages (**c**) and CD163^+^ macrophages (**d**) in tumour core and periphery in IL-1B^fl/fl^ and IL-1B^−/−^ mice. Data are mean ± SEM, Two-way ANOVA with Sidak’s post hoc test. **e**, **f** Quantification of CD34^+^ blood vessels and MPO^+^ neutrophils in IL-1B^fl/fl^ and IL-1B^−/−^ mice. Data are shown as mean +/− SEM, Two-tailed unpaired *t*-test. **g**, **h** Images and quantification of primary tumour development in IL1R1^fl/fl^ (*n* = 18 primary tumours from *n* = 9 mice) and IL1R1^−/−^ (*n* = 14 primary tumours from *n* = 7 mice) mice after intra-ductal administration of E0771 Luc2 V5 IL-1B-GFP. Normalised data are shown as mean +/− SEM, Two-tailed unpaired *t*-test.

After addressing the role of stromal-derived IL-1B signalling by using parental E0771 in mice lacking IL-1B or IL1R1, next we investigated whether tumour-derived IL-1B rescues the lack of microenvironment-derived IL-1B. For this, we engineered E0771 to overexpress IL-1B (Supplementary Fig. 2A, C) and performed an orthotopic engraftment in IL-1B^fl/fl^ and IL-1B^−/−^ mice. We found that in IL-1B^−/−^ mice there was a significant reduction in tumour growth compared to IL-1B^fl/fl^ mice (Fig. 3a, b) (for comparison the injection of the parental cell line can be observed in Fig. 1c, d). As tumour-derived IL-1B compensated for the absence of microenvironment-derived IL-1B, we next hypothesised that IL-1B production by tumour cells restores the infiltration of innate immune cells that may harbour anti-tumour functions, resulting in a reduction in primary tumour growth. Therefore, we quantified macrophages and neutrophils in primary tumours overexpressing IL-1B from IL-1B^fl/fl^ and IL-1B^−/−^ mice using IHC. We found an increase in F4/80^+^ macrophages in tumours growing in IL-1B^−/−^ mice in the periphery of the tumour compared to the core of the tumour (Fig. 3c). However, no differences were observed in primary tumours growing in IL-1B^fl/fl^ mice, suggesting that tumour-derived IL-1B induces F4/80^+^ macrophage recruitment (Fig. 3c). We also assessed the infiltration of CD163^+^ macrophages in primary tumours overexpressing IL-1B in IL-1B^fl/fl^ and IL-1B^−/−^ mice. We found an increase in CD163^+^ macrophages in the tumour periphery compared to the tumour core, suggesting increased macrophage recruitment (Fig. 3d). However, no significant difference was observed in CD163^+^ macrophages between tumour core and tumour periphery in IL-1B deficient mice (Fig. 3d). In line with impaired M2-like macrophage infiltration, we observed a decrease in CD34^+^ blood vessels in tumours growing in an IL-1B ablated microenvironment (Fig. 3e). In agreement with our hypothesis that tumour-derived IL-1B drives an anti-tumour response, we quantified the infiltration of neutrophils in primary tumours and found a tendency for an increase in MPO^+^ neutrophils (Fig. 3f). Next, we orthotopically engrafted IL-1B overexpressing E0771 in IL1R1^fl/fl^ and IL1R1^−/−^ mice and hypothesised that IL-1B does not trigger an immune response in an IL1R1 deficient microenvironment. We found that the growth of IL-1B overexpressing primary tumours was increased in IL1R1^−/−^ mice compared to IL1R1^fl/fl^ mice (Fig. 3g–h), suggesting that tumour-derived IL-1B can no longer exert its anti-tumour functions in IL1R1-deficient microenvironments. In conclusion, these data suggest that tumour derived IL-1B activates the innate immune response with potential anti-tumour functions. Both tumour-derived and microenvironment-derived IL-1B promote the infiltration of immune cells that may exert anti-tumour functions, impairing the growth of breast cancer at the primary site.

### Tumour-derived IL-1B signalling supports breast cancer metastasis by enhancing tumour cell motility and inhibiting cell proliferation

**Fig. 4:**
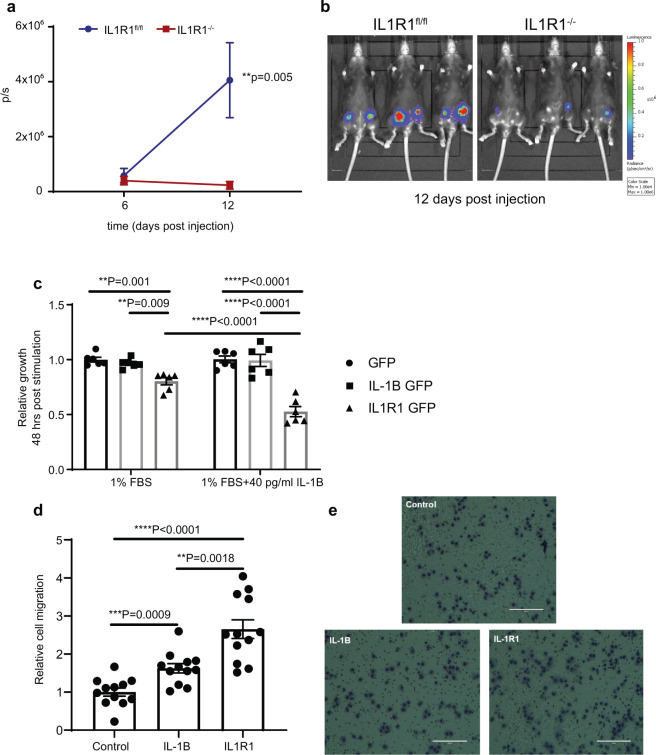
IL-1B signalling enables breast cancer metastasis initiation. **a**, **b** Tumour proliferation after injection of E0771 luc2 V5 IL1R1-GFP cells in IL1R1^fl/fl^ (*n* = 8 primary tumours from *n* = 4 mice) and IL1R1^−/−^ (*n* = 6 primary tumours from *n* = 3 mice) mice. Data are mean +/− SEM, Two-way ANOVA with Sidak’s multiple comparisons test. **c** In vitro relative tumour growth of E0771 luc2 V5 GFP, IL-1B GFP or IL1R1 GFP cells upon stimulation with 40 pg/ml mouse recombinant IL-1B. Data are mean (+/− SEM), Two-way ANOVA with Sidak’s multiple comparison test (*n* = 6 technical repeats from two biological replicates). **d** Transwell cell migration of IL-1B overexpressing (*P* = 0.0009) and IL1R1 overexpressing (*P* < 0.0001) E0771 luc2 V5 cancer cells compared to control GFP-expressing cells (no treatment with exogenous IL-1B). Comparison of cell migration in vitro between E0771 luc2 V5 IL-1B GFP and IL1R1 GFP tumour cells (*P* = 0.0018). Data are mean (+/− SEM) cell number from 4 fields of view derived from three biological experiments (*n* = 12 technical repeats). Two-tailed unpaired *t*-test with Welch’s correction. **e** Representative images of haematoxylin-stained control, IL-1B and IL-1R1 overexpressing cells migrated through the membrane. Scale bar = 200 µm.

We and others have previously shown that tumour-derived IL-1B drives EMT^11,14^. We suggested that inhibition of IL-1B using Canakinumab supports an epithelial rather than a mesenchymal phenotype thus, favoring tumour proliferation while inhibiting EMT and therefore metastasis^11^. In order to address the role of tumour-derived IL-1B signalling in an immune-competent syngeneic model, we orthotopically injected IL1R1-overexpressing E0771 (Supplementary Fig. 2B, D) mouse mammary tumour cells in IL1R1^fl/fl^ and IL1R1^−/−^ mice. In this model, IL-1B signalling is only active in tumour cells. We found that tumour-derived IL1R1 signalling decreased primary tumour growth in microenvironment of IL1R1 deficient mice compared to control IL1R1^fl/fl^ mice (Fig. 4a, b) (for comparison the injection of the parental cell line can be observed in Fig. 1a, b). Moreover, using an MTT proliferation assay we found that tumour cells overexpressing IL1R1 displayed reduced proliferation compared to control cells when stimulated with exogenous IL-1B (Fig. 4c). In contrast, E0771 cells overexpressing IL-1B or IL1R1 were found to be more invasive than control cell line. Moreover, E0771 cells overexpressing IL1R1 were significantly more invasive than cells overexpressing IL-1B (Fig. 4d, e). Taken together, these data suggest that tumour-derived IL-1B signalling, via IL1R1 activation, inhibits primary tumour growth, while enabling tumour invasion.

### Microenvironment-derived IL-1B enhances breast cancer metastasis in bone

**Fig. 5:**
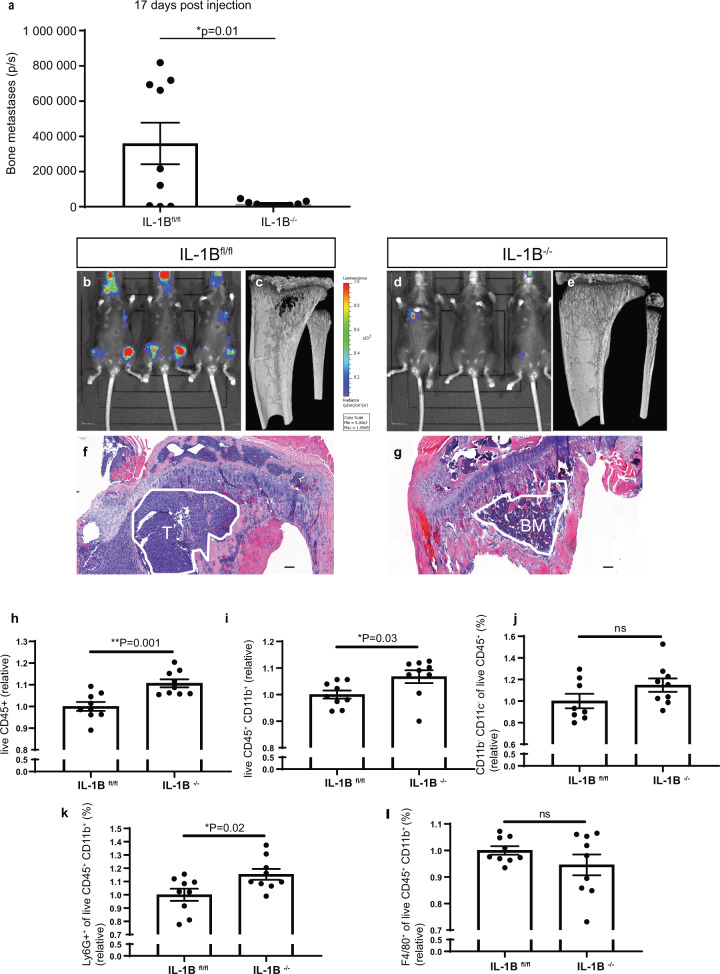
Microenvironment-derived IL-1B drives breast cancer bone metastases by controlling myeloid cell abundance. **a** Quantification (photons/seconds) of bone metastases in IL-1B^fl/fl^ and IL-1B^−/−^ mice after intra-cardiac administration of E0771 luc2 V5 GFP tumour cells. **b** Representative bioluminescence micrographs of metastases in bone and distant locations in IL-1B^fl/fl^ mice. **c** µCT scans and **f** H&E staining of tibia in tumour-bearing IL-1B^fl/fl^ mice. Scale bar = 100 µm. **d** Representative bioluminescence micrographs of metastases in bone and distant locations in IL-1B^−/−^ mice. **e** µCT scans and **g** H&E staining of tibia in tumour-bearing IL-1B^−/−^ mice. H&E images: BM bone marrow, T tumour. Data are mean ± SEM, Two-tailed unpaired *t*-test with Welch’s correction. Relative percentage of **h** immune cells (CD45^+^), **i** myeloid cells (CD45^+^CD11b^+^), **j** lymphoid cells (CD45^+^ CD11b^−^ CD11c^−^), **k** neutrophils (CD45^+^CD11b^+^Ly6G^+^) and **l** macrophages (CD45^+^ CD11b^+^ F4/80^+^) in bone marrow samples from femurs of IL-1B^fl/fl^ (*n* = 9) and IL-1B^−/−^ (*n* = 9) mice detected using multicolour flow cytometry. Data are mean ± SEM, Two-tailed unpaired *t*-test.

To investigate the effects that microenvironment-derived IL-1B has on breast cancer bone metastasis, E0771 mammary cancer cells were injected into the blood circulation of IL-1B^fl/fl^ and IL-1B^−/−^ mice via intra-cardiac injection. Bone metastasis, as quantified using bioluminescence, was significantly reduced by 95% in IL-1B^−/−^ mice (1.7 × 10^4^ p/s) compared to the IL-1B^fl/fl^ group (3.6 × 10^5^ p/s, *P* = 0.0196) (Fig. 5a, b, and d). In the IL-1B^−/−^ group, 2/9 (22.2%) mice developed bone metastasis, compared to 6/9 (66.7%) mice within the IL-1B ^fl/fl^ group. Osteolytic metastases were observed in IL-1B^fl/fl^ mice using µCT imaging (Fig. 5c), whereas osteolytic lesions were absent in IL-1B^−/−^ mice (Fig. 5e). No significant difference was found in osteoclast and osteoblast activity, when IL-1B^fl/fl^ mice injected with tumour cells were compared with IL-1B^−/−^ (Supplementary Fig. 3). Overt metastases replaced part of the tibial bone marrow in IL-1B^fl/fl^ (Fig. 5f), which was still present in IL-1B^−/−^ mice (Fig. 5g), based on H&E staining. Next, we monitored innate immune cells at this site using multi-colour flow cytometry. We found that a decrease in bone metastases upon depletion of microenvironment-derived IL-1B was accompanied by a significant increase in the proportion of CD45^+^ immune cells (Fig. 5h). The increase in CD45^+^ immune cells was accompanied by an increase in CD45^+^ CD11b^+^ myeloid cells (Fig. 5i), whereas there was no difference in CD45^+^ CD11b^−^ CD11c^−^ lymphoid cells (Fig. 5j). The increase in CD45^+^ CD11b^+^ myeloid cells was reflected by an increase in CD45^+^ CD11b^+^ Ly6G^+^ neutrophils (Fig. 5k), whereas no difference in CD45^+^ CD11b^+^ F4/80^+^ macrophages was observed (Fig. 5l). Together, these data suggest that microenvironment-derived IL-1B promotes breast cancer bone metastasis in vivo. Because IL-1B displays opposite functions in primary breast tumour and bone metastasis, its role in metastatic breast cancer may be tissue-specific.

### Combining Anakinra with Doxorubicin and Zoledronic acid inhibits breast cancer growth at the primary site and distant recurrence, in pre-clinical models of breast cancer metastasis

**Fig. 6:**
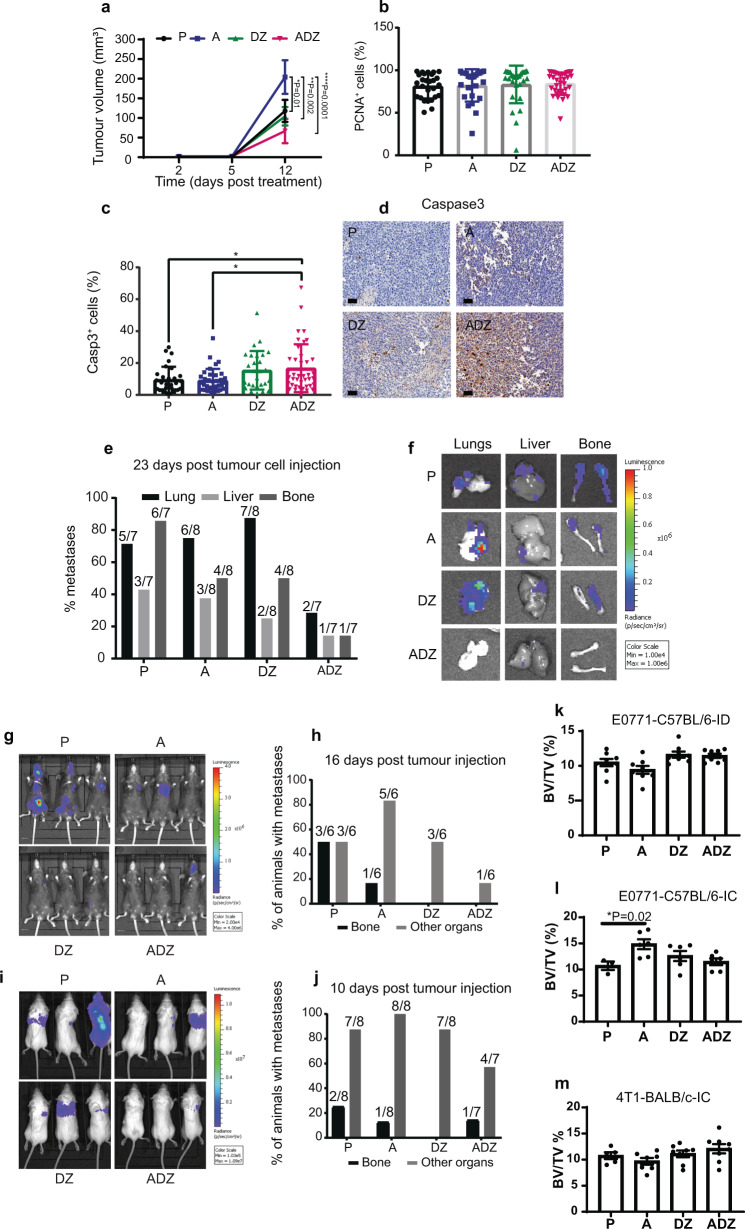
Combining Anakinra with Doxorubicin and Zoledronic acid reduces primary tumour growth, distant recurrence and bone metastasis in breast cancer. **a** Primary tumour volume in Placebo (P) (day 2: *n* = 11 tumours (*n* = 8 mice), day 5: *n* = 11 tumours (*n* = 7 mice), day 12: *n* = 13 tumours (*n* = 7 mice)), Anakinra (A) (day 2: *n* = 9 tumours (*n* = 7 mice), day 5: *n* = 9 tumours (*n* = 6 mice), day 12: *n* = 13 tumours (*n* = 8 mice), Dox and Zol (DZ) (day 2: *n* = 8 tumours (*n* = 6 mice), day 5: *n* = 15 tumours (*n* = 8 mice), day 12: *n* = 15 tumours (*n* = 8 mice)), Anakinra, Dox, and Zol (ADZ) (day 2: *n* = 8 tumours (*n* = 5 mice), day 5: *n* = 13 tumours (*n* = 8 mice), day 12: *n* = 13 tumours (*n* = 7 mice)). Data are mean +/− SEM, two-way ANOVA with Tukey’s multiple comparisons test (A vs. P *P = 0.01, A vs. DZ **P = 0.002, A vs. ADZ ***P = 0.0001). **b**, **c** PCNA^+^ and Cleaved-Caspase 3^+^ cells (%) in primary tumours. Each data point represents one or more tissue sections from an individual primary tumour. Data are mean +/− SD. One-way ANOVA with Tukey’s multiple comparisons test (P vs. ADZ *P = 0.04, A vs. ADZ *P = 0.02). Scale bar = 50 µm. **d** Casp3^+^ IHC. **e**, **f** Metastases (%) and BLI images (P (*n* = 7 mice), A (*n* = 8 mice), DZ (*n* = 8 mice), or ADZ (*n* = 7 mice)). Fisher’s exact test: *P = 0.02 P vs. ADZ (bone metastases); *P = 0.04 DZ vs. ADZ (lung metastases). **g**, **h** Animals with metastases (%) (P (*n* = 6), A (*n* = 6), DZ (*n* = 6), or ADZ (*n* = 6)) (intra-cardiac injection of E0771 luc2 GFP cells). **i**, **j** Animals with metastases (%) P (*n* = 8), A (*n* = 8), DZ (*n* = 8), or ADZ (*n* = 7) (intra-cardiac injection of 4T1 luc2 cells). **k**–**m** BV/TV % in tibiae from the spontaneous metastasis (**k**) and overt bone metastasis (L-C57BL/6, M-BALB/c) models.

Our data show that IL-1B drives the recruitment of innate immune cells, and whilst IL-1B is protective in the primary tumour, it drives metastasis in bone. We, therefore, investigated whether targeting IL-1B in combination with standard of care agents that have immune-modulatory effects impairs primary tumour growth as well as metastasis. For this purpose, we used IL-1 receptor antagonist, Anakinra, an anti-inflammatory drug, in combination with Doxorubicin (Dox) and Zoledronic acid (Zol), both pro-inflammatory, in a syngeneic pre-clinical in vivo model of spontaneous breast cancer metastasis. Bone homing mouse mammary tumour cells (E0771) were orthotopically engrafted in 6–8 week-old C57BL/6 female mice. Nine days after tumour cell injection, mice were randomised to the following groups: placebo (P) (*n* = 8) (daily by subcutaneous injection), Anakinra (*n* = 8) (daily by subcutaneous injection), Dox (weekly by intra-venous injection) followed 24 h later by Zol (*n* = 8) (weekly by subcutaneous injection), and the combination treatment Anakinra, Dox, and Zol (*n* = 8). We found that Anakinra alone significantly increased the growth of primary tumours, however, adding Dox and Zol to Anakinra significantly decreased primary tumour growth, compared to Anakinra alone, (12 days of post-treatment) (Fig. 6a), without affecting cell proliferation (Fig. 6b) but by inducing cell apoptosis (Fig. 6c, d).

Using the same cohort of animals, we then investigated the effect of the single and combined treatments on metastases in bone and other organs (lungs and liver), in the spontaneous metastasis model. Anakinra alone reduced the percentage of animals showing metastasis in bone (50% compared to 85.71% in the placebo treated group) and liver (37.5% vs. 42.86% in the placebo). Similarly, Dox and Zol alone treatment resulted in a reduction in bone (50% vs. 85.71% in the placebo) and liver metastasis (25% vs. 42.86% in the placebo). However, both Anakinra alone and Dox and Zol alone caused an increase in the percentage of animals with lung metastasis (75% Anakinra, 87.5% Dox and Zol, and 71.43% in placebo). Combining Anakinra with Dox and Zol reduced metastasis in lungs (28.57% vs. 71.43% in the placebo), liver (14.29% vs. 42.86% in the placebo) and significantly impaired metastasis in bone (14.29% vs. 85.71% in the placebo) (Fig. 6e, f).

We next investigated the effect of combining Anakinra with Dox and Zol on outgrowth of disseminated breast cancer cells in metastatic organs. For this, immunocompetent 6–8 week-old C57BL/6J or BALB/c female mice received an intra-cardiac injection of luciferase expressing E0771 or 4T1 mouse mammary tumour cells, respectively and the development of metastasis was monitored using in vivo bioluminescence imaging. Mice were randomised into the following treatment groups 2 days after tumour cell injection: placebo, Anakinra, Dox and Zol and the combination of Anakinra, Dox and Zol. In the E0771/C57BL/6J model, in vivo bioluminescence imaging of bone, liver, and lungs was performed in end-stage mice 16 days of post-treatment. Our data showed that the percentage of animals showing bone metastasis was reduced upon Anakinra (16.67% vs. 50% in the placebo) or Dox and Zol treatment (0% vs. 50% in the placebo). However, the percentage of animals showing metastatic outgrowth in other sites was increased with Anakinra treatment (83.33% vs. 50% in the placebo), but remained unaltered in the Dox and Zol group (50% following Dox and Zol). Importantly, combining Anakinra with Dox and Zol resulted in a striking reduction in both bone and distant metastasis (overt metastases in bones from 0% of animals and in other organs from 16.67% of animals) (Fig. 6g, h). In confirmation of our findings, we did not observe a difference in bone metastasis when comparing the treatments Anakinra, Anakinra+Zol, and Anakinra+Dox (Supplementary Fig. 4). Therefore, we combined Anakinra with Dox+Zol and concluded that only triple treatment is superior in reducing tumour growth in both bone and soft tissue.

In the 4T1/BALBc model, 25% of animals in the placebo group developed bone metastasis, compared to 12.5% in the Anakinra group whereas no bone metastases were observed after Dox and Zol treatment. Combining Anakinra to Dox and Zol resulted in a reduction in bone metastasis compared to placebo, similarly to the Anakinra treatment alone. The combination treatment also reduced metastasis elsewhere (57.1% of the animals developed metastasis compared to 87.5% in the placebo treated group) (Fig. 6i, j). The combination treatment did not display damaging effects on bone formation in any of the pre-clinical models used in this study (Fig. 6k–m). Hence, combining IL-1B signalling inhibition with standard of care is a promising treatment for breast cancer bone metastasis.

### Combining Anakinra with Doxorubicin and Zoledronic affects primary tumour immune cell composition

**Fig. 7:**
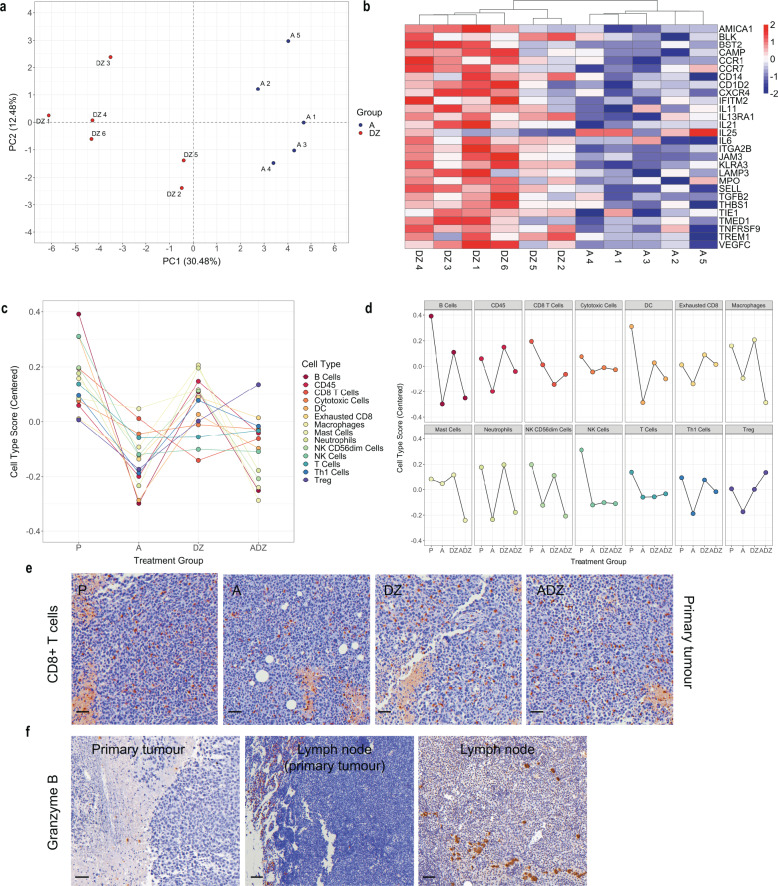
Combining Anakinra, Doxorubicin, and Zoledronic acid affects tumour-associated immune signatures in a syngeneic model of spontaneous breast cancer metastasis to bone. **a** PCA plot. Each dot corresponds to a primary tumour from each treatment group (A or DZ). **b** Hierarchical clustering of the 28 differently regulated genes between primary tumours treated with A (*n* = 5 primary tumours from five animals) and DZ (*n* = 6 primary tumours from six animals). **c** Single and **d** facet plot of immune cell type score in primary tumours developed in C57BL/6 mice orthotopically inoculated with E0771 luc2 GFP and treated with A, DZ, ADZ, or control (P). **e** Representative immunohistochemistry images of CD8^+^ T cells in primary tumours of end-stage mice treated with P, A, DZ, or ADZ. Data are mean +/− SEM, One-way ANOVA with Tukey’s post-hoc test, ns. **f** Representative immunohistochemistry images of Granzyme B^+^ cells in primary tumour from control mice, corresponding lymph node and lymph node from tumour-naïve mice. Scale bar = 50 µm.

Since the chosen treatments, both alone and in combination, altered the immune response, we used NanoString amplification-free gene expression profiling technology to identify the immune signature of breast primary tumours and the subsequent development of distant metastasis in bone and other organs. Using the NanoString nCounter™ panCancer mouse Immune Profiling Panel, we found that, amongst an expected enrichment in immune cell pathways, cytokine production, cytokine–cytokine receptor interaction, cell chemotaxis, and specifically myeloid leucocyte migration were enriched (Supplementary Fig. 5). Anakinra treatment caused opposite effects on immune response compared to Dox and Zol. The PCA plot and the hierarchical cluster analysis showed that an anti-inflammatory signature was observed after Anakinra treatment compared to Dox+Zol (Fig. 7a, b). Cell signature analysis demonstrated reduced abundance of every immune cell type, such as B cells, T cells (including CD8+ T cells, Th1 cells, exhausted CD8+ T cells and immunoregulatory (Treg) cells, dendritic cells (DCs), macrophages, mast cells, neutrophils and NK cells, including, CD56dim (‘cytotoxic’) NK cells compared to control group following Anakinra treatment (Fig. 7c, d). The anti-inflammatory effect of Anakinra was maintained in the combination treatment with Dox+Zol, resulting in a reduced immune cell abundance. Interestingly, macrophages and mast cells were further reduced upon triple treatment compared to any other treatment group, in contrast, T regulatory cells were increased. As the combination treatment significantly reduced primary tumour growth and distant recurrence, while causing an increase in Treg cells alongside a decrease in immune cells with cytotoxic functions, including CD56dim (cytotoxic) NK cells and CD8+ (cytotoxic) T cells, we suggest that an anti-tumour immune response may have been induced. In support of the cell signature analysis, IHC for CD8+ T cells and Granzyme B demonstrated CD8+ T cell infiltration of primary tumours, but that exhaustion had already taken place (i.e., an absence of Granzyme B+ cells) (Fig. 7e, f). IHC for F4/80, CD163, and MPO was performed and a significant increase in MPO+neutrophils was found in primary tumours upon Dox+Zol treatment compared to any other treatment (Supplementary Fig. 6). Based on the cell signature plots, Anakinra exerted an anti-inflammatory effect, by reducing the abundance of every immune cell type. Taken together, these data suggest that the specific combination treatment is effective at reducing primary tumour growth and distant recurrence in bone and other organs by affecting the immune response.

## Discussion

Herein, we show that microenvironment-derived IL-1B has opposite function at different sites: IL-1B supports the development of breast cancer bone metastasis, whilst also reducing tumour growth at the primary site. These results are concordant with our previous findings on the pharmacological inhibition of IL-1B signalling by repurposing Anakinra or Canakinumab, which is already approved for the treatment of auto-inflammatory diseases^11^. Inhibition of IL-1B signalling has been shown to have an anti-tumour effect in the CANTOS trial, in that patients with previous myocardial infarction and high C-reactive protein levels assigned to receive Canakinumab, displayed reduced lung cancer mortality^31,32^.

Here, we suggest that increased primary tumour growth upon IL-1B inhibition in the microenvironment is associated with microenvironment-derived IL-1B promoting the infiltration of innate immune cells that may display anti-tumour functions. These results align with data published by others, which demonstrate that inhibiting IL-1B signalling using the IL1R1 receptor antagonist reduces myeloid cell accumulation at the primary tumour site in E0771 syngeneic murine models^33^. Similarly, IL-1B has been found to promote the infiltration of monocytes and their differentiation into macrophages within the primary tumour^19^. In support of our findings that IL-1B drives the infiltration of innate immune cells with putative tumour-killing capacity, by inhibiting M2-like macrophages, we found a reduction in CD34^+^ blood vessels in tumours overexpressing IL-1B. We also observed that IL-1B promotes macrophage recruitment to the primary tumour site, potentially controlling macrophage polarisation and localisation. However, in contrast with our findings, macrophages recruited to the primary tumour have been described to differentiate towards a tumour-supporting phenotype^19^. We suggest that the use of different murine strains might explain this divergence. In melanoma models, IL-1B has been shown to increase the anti-tumour potential of T helper 1 (Th1) cells^34^. We assessed the infiltration of CD8+ T cells in primary tumours from wild-type and IL-1B overexpressing E0771 tumour cells in IL-1B^fl/fl^ and IL-1B^−/−^ microenvironments. We observed a shift in T cell infiltration (Supplementary Fig. 7). Interestingly, CD8^+^ T cell infiltration upon injection of IL-1B overexpressing E0771 cancer cells mirrored the infiltration pattern of F4/80^+^ macrophages. We speculate that this may further support our hypothesis that tumour-derived IL-1B restores an anti-tumour immune response. However, these trends were not statistically significant, hence the role of the adaptive immune system in our models remains to be determined. It is also important to consider that the in vivo models used in this work allow for a rapid development of primary tumour growth and bone metastasis, and therefore do not take into account the role of IL-1B in the context of chronic inflammation that associates with tumour development. To our knowledge, there are currently no immune competent mouse models that enable us to investigate effects of IL-1B on the indolent stage of bone metastasis or effects on slow growing tumours. However, our previously published data, using T cell deficient mice, have demonstrated that inhibiting IL-1B or IL1R signalling can both hold disseminated tumour cells in a dormant state in bone preventing metastatic outgrowth and slow down growth of established tumours in bone^13,15^. It should be noted that, our work does not take into account IL-1A. Indeed, IL1R1 can be activated by both IL-1B and IL-1A. In a transgenic model of luminal breast cancer, IL-1A is tumour-suppressive and correlates with better prognosis in patients^35^. A similar function for IL-1A and IL-1B has also been reported: IL-1A and IL-1B produced by breast cancer cells can display the same function by equally activating IL1R1 in lung fibroblasts, which support metastatic colonisation^36^. Further work is required to investigate the functional significance of this IL-1 family member in bone metastasis.

Our previous work suggests that IL-1B drives breast cancer metastasis in humanised models by inducing EMT at the primary tumour^11^. In line with these results, we found that overexpression of IL-1B signalling in murine mammary tumour cells reduced proliferation and increased migration in vitro, supporting the hypothesis that IL-1B drives EMT. In line with our results, immune cell-derived IL-1B promotes a mesenchymal phenotype in metastasis initiating cells and prevents metastatic colonisation, reducing the outgrowth of distant metastases^14^. However, only lung metastasis was investigated in this study, and IL-1B may have a different effect on MIC colonisation within bone.

In bone metastasis, IL-1B displays a tumour-supportive function and may control myeloid cell number. We speculate that in response to cancer-associated inflammation at the primary site, IL-1B is produced systemically and drives the mobilisation of immune cells from the bone marrow. This has the potential to reduce the number of immune cells patrolling bone, hence supporting the development of bone metastasis. In particular, in contrast to the primary tumour site, we found a decrease in neutrophil number. It has been proposed that IL-1B promotes the recruitment of anti-tumour neutrophils to the lung metastatic niche, consequently reducing metastasis^37^. This implies that IL-1B may have different effects on neutrophil recruitment in bone versus other sites, which is concordant with our data that microenvironment-derived IL-1B promotes the recruitment of anti-tumour neutrophils to the primary tumour site, but reduces recruitment to bone. However, published studies have demonstrated that microenvironment-derived IL-1B produced by macrophages induces the mobilisation of a different subset of neutrophils, with an immunosuppressive and pro-metastatic phenotype^16^ and that, in turn, extracellular traps produced by neutrophils induce IL-1B production by macrophages in inflammatory diseases^38,39^. It is to be considered that in the bone marrow of IL-1B^−/−^ mice the lack of bone metastases may be responsible for the increase of neutrophils. In cancer, neutrophils have been reported to have both anti-tumour and pro-tumour functions. The state of maturation of neutrophils can be different depending on the tissue, where they are located and their function in cancer can be different depending on their maturation state^40,41^. We used different markers to identify neutrophils, hence microenvironment-derived IL-1B may differentially regulate the recruitment of different subsets of neutrophils at different sites. In our model, IL-1B drives the recruitment of innate immune cells that may elicit an anti-tumour response, irrespective of the source. Both tumour and microenvironment-derived IL-1B induce the recruitment of immune cells with a pro-inflammatory, anti-tumour profile. Establishing the function of these immune cells requires future investigations.

The role of IL-1B in cancer is controversial. Although our work suggests that IL-1B drives the infiltration of innate immune cells with potential anti-tumour functions, others have found that IL-1B creates an immunosuppressive microenvironment that facilitates distant metastasis by enhancing the infiltration of myeloid-derived-suppressor cells, which suppress cytotoxic T cell and DC activity^19,42^. Hence, coupling anti-IL-1B treatment with immunotherapies could be beneficial. Current clinical trials are recruiting patients to investigate the safety and efficacy of the IL-1B antibody, Canakinumab, alongside immunotherapies such as the immune checkpoint inhibitors Spartalizumab and Pembrolizumab in breast cancer and other tumour types (ClinicalTrials.gov Identifier: NCT03484923, NCT03631199, NCT03968419, and NCT03742349). However, the function of IL-1B as a critical inflammatory cytokine driving the immune response to pathogens, and the risks associated with inhibiting its function in patients should be taken into account. Additionally, our data suggest that although inhibition of IL-1B has the potential to reduce bone metastasis, primary tumour growth may increase. Surgical resection of the primary tumour alongside IL-1B targeting may be appropriate.

Breast cancer patients with bone metastasis are commonly treated with the anti-resorptive agent Zol. Clinical trials have shown that adjuvant Zol in combination with chemotherapy reduces bone metastasis in patients with early breast cancer^25^. However, an increase in metastasis in other sites has been observed in pre-menopausal women receiving this treatment regime^25,43^. In addition, in line with other published reports^33^, Anakinra treatment, when administered alone, may enhance non-skeletal metastasis. Here, we found that combining standard of care with Anakinra reduces the growth of the primary tumour, bone metastasis as well as recurrence in other organs. Importantly, lung metastasis was significantly reduced upon combination treatment compared to Dox+Zol. Studies showing the use of Anakinra after administration of chemotherapy (Paclitaxel) have reported a slight reduction in primary tumour growth and an increase in lung metastasis^33^. This data may suggest that the timing of exposure to anti-IL1R1 treatment as well as the type of chemotherapy administered may play critical roles in the anti-tumour efficacy when combining with Anakinra. Doxorubicin is a known inducer of immunogenic cell death^44^, Zol has been reported to display pro-inflammatory functions^45,46^, whilst Anakinra is a well-known anti-inflammatory drug^47^. Interestingly, Zol has been reported to boost the immunogenicity of other forms of cell death, which are generally non-immunogenic, therefore boosting the host immune response against cancer^48^. By inhibiting IL-1A and IL-1B binding to IL1R1, Anakinra reduces the immunosuppression caused by myeloid-derived suppressor cells and impairs autoinflammation caused by IL-1A-dependent IL-1B stimulation^49,50^. Considering the immune-modulatory effects of each treatment, we sought to understand whether the combination treatment alters the immune response. We found that Anakinra displays an opposite immune-related signature compared to Dox and Zol. We suggest that Dox and Zol induce a pro-inflammatory anti-tumour response, that although initially beneficial, can lead to the onset of immunosuppression due to sustained inflammation and the development of a pro-tumour response. Hence, adding an anti-inflammatory drug like Anakinra is needed to halt this transition and rebalance an effective immune response against metastatic breast cancer. The combination therapy initiates a pro-inflammatory anti-tumour response that is subsequently exhausted (no detectable Granzyme B^+^ cells and influx of Treg cells) in mammary tumours. Combining Anakinra with standard of care Dox+Zol in pre-menopausal women may be beneficial in reducing the immune-suppressive effect of Oestrogen whilst boosting the anti-tumour effect of Dox+Zol. Oestrogen and Zol have opposite effects on the infiltration and action of T regulatory cells and polarisation of macrophages, with oestrogen interfering with Zol-driven anti-tumour immune response. This potentially explains the differential effect of Zol in pre-menopausal and post-menopausal women. We are currently investigating molecular mechanisms underpinning the differential effects of Zol in pre-menopausal and post-menopausal women^51^.

Here, we propose that combining Anakinra with standard of care treatment affects the immune response and as a result reduces primary tumour growth, bone metastasis and recurrence in other organs.

## Methods

### Animals

In vivo experiments were performed using 6–8-week-old female IL-1B^fl/fl^^52^ (control) or IL-1B^−/−^ mice (C57BL/6J background), IL1R1^fl/fl^ (control)^27^, IL1R1^−/−^ mice (C57BL/6J background)^27^, C57BL/6J and BALB/c. Ubiquitous knockout for IL-1B was obtained by backcrossing PGK;Cre; IL-1B^fl/fl^ to IL-1B^fl/fl^ mice (Supplementary Fig. 1). The knockout was confirmed by genotyping using the following primers:

PGK:Cre:

5′-GCATTACCGGTCGATGCAACGAGTGATGAG-3′,

5′-GAGTGAACGAACCTGGTCGAAATCAGTGCG-3′,

5′-GGACAGGACTGGACCTCTGCTTTCCTAGA-3′,

5′-TAGAGCTTTGCCACATCACAGGTCATTCAG-3′

IL-1B^fl/fl^:

GenoF1: TGTTGGGTGATCTCCGTTGA/GenoR1: TCTCCACAGCCACAATGAGT

IL-1B^−/−^:

Geno F1: TGTTGGGTGATCTCCGTTGA/Geno R2: CCCTGGCTGCTTTTATGACT.

Genotyping protocol for PGK:Cre and IL-1B were kindly provided by Dr. Allan Lawrie (University of Sheffield) and Dr. Emmanuel Pinteaux (University of Manchester), respectively. Genotyping was performed by BioServ UK Ltd. Reduced *Il-1b* gene expression in IL-1B^−/−^ mice was confirmed using real-time PCR (Supplementary Fig. 1C). Briefly, RNA was extracted from lung tissues of IL-1B^fl/fl^ and IL-1B^−/−^ naive mice. Lungs were homogenised in TRI reagent followed by centrifugation in QIAshredder columns (QIAGEN). Aqueous solution containing RNA was obtained by adding chloroform and RNA precipitated in isopropanol, according to the TRI reagent® protocol (Sigma-Aldrich). cDNA synthesis was performed using LunaScript RT SuperMix Kit following manufacturer’s instructions (New England BioLabs). Real-time PCR was performed using Taqman universal PCR master mix (4304437, Applied Biosystems) and 7900HT PCR system (Applied Biosystems) (Genomic Core Facility, University of Sheffield). To measure *Il-1b* expression, Taqman probe, Mm00434228_m1(ThermoFisher) was used. GAPDH and B-actin were used as housekeeping genes (GAPDH: Mm99999915_g1, B-actin: Mm02619580_g1, ThermoFisher). Fold change in gene expression was calculated using the ΔΔCT method. IL1R1^fl/−^ were kindly provided by Dr. Emmanuel Pinteaux (University of Manchester). IL1R1^fl/fl^ and IL1R1^−/−^ were obtained by IL1R1^fl/-^ incross and genotypes confirmed as previously described^27^. Food and water were provided ad libitum. Mice were maintained on a 12 h light:12 h dark cycle. In vivo procedures were conducted in accordance with local guidelines and with UK Home Office approval under Project License (PPL) 70/8964 or P99922A2E, University of Sheffield, UK.

#### Cell lines and in vitro studies

Murine mammary tumour E0771 Luc2 V5 GFP, E0771 Luc2 V5 IL-1B GFP, and E0771 Luc2 V5 IL1R1 cell lines were generated using lentiviral transduction (Supplementary Fig. 2). The following plasmids were used for overexpression studies: IL-1B (MR226719L4, Origene), IL1R1 (MR227508L4, Origene), GFP (17448 pLenti CMV GFP Puro (658-5), Addgene), Luciferase (21474 pLenti CMV V5-LUC Blast (w567-1), Addgene). For lentiviral transduction, HEK293T cells were transfected with one of the above expression vectors and packaging plasmids pMD2.G and psPAX2, kindly provided by R. Bishop (University of Sheffield). Twenty-four to forty-eight hours of post-transfection, viral particles were collected, filtered (0.45 µm) and stored at −80 °C. Virus concentration was measured using Lentivirus-Associated p24 ELISA Kit (VPK-107, Cell Biolabs). E0771 cells were seeded in a 24-well plate (2.5 × 10^4^/well) and lentivirus particles (2 moi) added with polybrene for 24 h. Puromycin (2 µg/ml) or Blasticidin (20 µg/ml) were used for selection. Overexpression of IL-1B and IL1R1 was confirmed via real-time PCR as previously described (Taqman probes: Mm00434228_m1 (*Il-1b*), Mm00434231_m1 (*Il1r1*), Mm99999915_g1 (*Gapdh*) and Mm02619580_g1 (*B-actin*)) and enzyme-linked immunosorbent assays (ELISA MAX^TM^ Deluxe Set Mouse IL-1B kit (BioLegend) and Mouse IL1R1 ELISA Antibody Pair Set (Sino Biological, SEK50807). Cell viability was assessed using MTT according to the manufacturer’s instruction (M5655-1G, Sigma-Aldrich). For cell migration, E0771 control, IL-1B overexpressing or IL1R1 overexpressing cells were incubated with 10 µg/ml Mitomycin C (Sigma-Aldrich, M-0503) for 3 h. Cells were collected, washed in PBS and resuspended in 0.1% w/v BSA RPMI 1640 (serum free). 1 × 10^5^ cells were added into the inner transwell (6.5 mm diameter inserts, 8 µl pore size, 3422, Costar) in a 24-well plate. Six hundred microliter complete medium was added to the main/outer well. Cell migration was quantified 24 h after seeding. Cells were fixed by washing the transwell membrane in 100% v/v ethanol (5 min) and stained with 1% w/v eosin (1 min) and haematoxylin solution (5 min). Membranes were mounted on slides using aqueous mounting solution (S3025, DAKO). Images of four different fields of view (FOV) were acquired from each cell line using Invitrogen^TM^ EVOS^TM^ FL auto imaging system (Thermo Fisher Scientific) (20× objective). Cell migration was quantified by image analysis using ImageJ.

Cell lines were cultured in RPMI 1640 Medium, GlutaMAX^TM^ (Gibco^TM^) containing 10% v/v foetal bovine serum (FBS). E0771 luc2 GFP and 4T1 luc2 were cultured in RPMI+10% v/v FBS (Gibco, Invitrogen). As described by ATCC, The E0771 mammary tumour was first reported in 1948 as a spontaneous mammary carcinoma arising in a C57Bl/6 mouse (https://www.lgcstandards-atcc.org/products/all/CRL-3461.aspx#generalinformation). E0771 luc2 GFP were kindly provided by Professor Sandra McAllister’s laboratory (Department of Medicine, Harvard Medical School, Boston, MA, USA). The E0771 cells used in the current study have been selected for their bone homing capabilities following two rounds of intra-cardiac tumour cell injection, in which bone metastatic clones have been isolated and re-injected. 4T1 luc2 were kindly provided by Ryan Bishop (Department of Oncology and Metabolism, University of Sheffield, UK).

All cell lines were cultured at 37 °C in a humidified incubator under 5% v/v CO_2_. Cells were routinely checked for mycoplasma infection.

### In vivo studies

#### Syngeneic model of primary tumour growth

12.5 × 10^4^ E0771 luc2 GFP, luc2 V5 GFP, IL-1B GFP, or IL1R1 GFP cells were engrafted via intra-ductal injection into IL-1B^fl/fl^, IL-1B^−/−^, IL1R1^fl/fl^, or IL1R1^−/−^ mice immediately following injection of 0.003 mg Vetergesic. Tumour growth was measured using bioluminescence-based in vivo imaging (IVIS Lumina II in Vivo Imaging System (PerkinElmer) and Living Image® 4.5.4 software), once per week, 2–4 min following subcutaneous injection of 6 mg/kg d-Luciferin (Invitrogen). BLI thresholds have been modified across experimental models to allow the visualisation of tumour-derived bioluminesce signal. Procedures were ended when tumour size reached 1 cm^3^ in either the control or treatment group and according to the original, planned, experimental protocol.

### Syngeneic model of experimental overt bone metastases

2.5 × 10^4^ E0771 luc2 V5 GFP, IL-1B GFP, or IL1R1 GFP cells were inoculated systemically via injection into the left cardiac ventricle of IL-1B^fl/fl^ or IL-1B^−/−^ mice. Tumour development in organs and bone was measured using bioluminescence imaging, as described above. Procedures were ended one week following detection of bone metastases.

#### Combination therapy

A syngeneic orthotopic model was used to study spontaneous breast cancer metastasis to bone. 1.25 × 10^5^ E0771 luc2 GFP were administered to 6–8 week-old C57BL/6J female mice via intra-ductal injection (4th mammary ducts). Mice were randomised 9 days after tumour cell injection to Placebo (P) (*n* = 8), Anakinra (*n* = 8), Doxorubicin and Zoledronic Acid (Dox and Zol) (*n* = 8) and Anakinra in combination with Dox and Zol (*n* = 8). Mice received a daily subcutaneous injection of Anakinra (1 mg/kg) or Placebo or intravenous 2 mg/kg/week Dox injection followed 24 h later by subcutaneous injection of Zol (100 µg/kg/week). Placebo and Anakinra (r-metHu1L-ra) were obtained from Amgen, Cambridge, UK. Dox and Zol were obtained from Pharmachemie B.V. and Novartis Pharma AG, respectively. Tumour growth was measured using bioluminescence-based in vivo imaging (IVIS Lumina II In Vivo Imaging System (PerkinElmer) and Living Image® 4.5.4 software) and using calipers. Mice were culled 23 days after tumour cell injection. Primary tumours were either fixed in 4% w/v PFA in phosphate buffered saline (PBS, PFA/PBS) or stored at −80% in FCS+10% v/v dimethyl sulfoxide (DMSO). Bones (hind limbs), lungs and liver were imaged ex vivo to detect metastasis.

In order to study overt bone metastasis, intra-cardiac injection of E0771 luc2 GFP (2.5 × 10^4^ cells) or 4T1 luc2 (0.5 × 10^5^ cells) was performed in 6–8 week old C57BL/6J or BALB/c (Charles Rivers), respectively. Mice were imaged using bioluminescence imaging before being randomised in the following treatment groups: Placebo (6–8 mice), Anankinra (6–8 mice), Dox and Zol (6–8 mice), Anakinra, Dox and ZOL (6–8 mice). Pharmacological treatments were started 3 days after tumour cell administration. Compound injections were performed as described for the spontaneous metastasis model. Metastasis formation was monitored using IVIS imaging, as described above. Mice were culled 16 days (E0771/C57BL/6J model) and 10–13 days after tumour cell injection (4T1/BALB/c model).

#### Sample collection

Primary tumours, tibiae, lungs, liver, and spleen were fixed in 4% w/v PFA/PBS for 48 h at 4 °C or stored at −80 °C in FBS+v/v 10% DMSO. Following blood collection, serum was obtained by centrifugation and stored at −80 °C. Bone marrow was collected from femurs via centrifugation and stored at −80 °C in 0.2 ml FBS+10% v/v DMSO.

#### Histology and immunohistochemistry

Fixed tissues were embedded in paraffin, sectioned at 3 µm and stained with H&E or used for immunohistochemistry (antibodies and antigen retrieval were performed as described in Supplementary Table 1). Briefly, paraffin-embedded sections were dewaxed and rehydrated. 3% v/v H_2_O_2_ in methanol was used to block internal peroxidase activity for 10 min, followed by antigen retrieval. Incubation with primary antibodies was overnight at 4 °C. Secondary antibodies were added for 30 min at room temperature (RT). ABC kit and DAB were used to amplify and visualise antibody binding prior to counterstaining with Gill’s Haematoxylin. Tissue sections were digitally scanned using Panoramic 250 Flash III slide scanner (3DHISTECH). Quantification of percentage of positive cells was performed using QuPath: Open source software for digital pathology image analysis^53^. Percentage of positive cells was calculated over the total number of cells (positive and negative for each cell marker assessed). These quantifications were performed considering the whole slide scanned.

#### Flow cytometry

Cryopreserved bone marrow was defrosted and washed in ice-cold PBS supplemented with 1% v/v FBS (FACS buffer). Samples were aliquoted and incubated with fluorochrome-conjugated antibodies and live/dead dye (Supplementary Table 2) (diluted 1:100) for 45 min on ice. After washing in FACS buffer, samples were resuspended in 500 µl FACS buffer. Data acquisition was performed on a BD LSRII™ flow cytometer and analysed using FlowJo, LLC. Gating strategy is reported in Supplementary Fig. 8.

#### µCT imaging

µCT analysis was carried out using a Skyscan 1172 X-ray–computed µCT scanner (Skyscan) equipped with an X-ray tube (voltage, 49 kV; current, 200 mA) and a 0.5-mm aluminium filter. Pixel size was set to 4.3 µm and scanning initiated from the top of the proximal tibia as described previously^22,24^.

#### NanoString nCounter™ gene expression profiling

To assess the immune profile of primary tumours upon treatment, the NanoString nCounter™ panCancer Immune profiling panel (NanoString Technologies) was used. Primary tumours were homogenised using QIAshredder (Catalogue number: 79654, Qiagen) and RNA isolated using RNeasy mini kit (Cat number: 74101, Qiagen). One hunderd and fifty nanogram RNA (50 ng/µl) was used for NanoString analysis, according to the manufacturer’s recommended protocols. Transcriptomic analysis was performed using nSolver^TM^ 4.0 and Advanced Analysis 2.0 (NanoString Technologies). Principal component analysis and differential gene expression testing between primary tumours treated with Anakinra or Dox and Zol was performed using Qlucore Omics Explorer. GO analysis was performed on differentially expressed genes in the Anakinra and Dox and Zol treatment groups, using g: Profiler (version e99_eg46_p14_f929183)^54^ and Metascape^55^. Genes were considered differentially expressed after setting *P*-value and FDR < 0.05 and absolute fold change ≥2. Cell-type deconvolution across treatment groups was performed in R; gene expression intensities were mean-centred across all samples before calculating raw cell-type signature scores as the mean average of log_2_ expression intensities for all genes in each cell-type signature. Signatures genes for each cell type were determined with reference to Nanostring document LBL-10043-08 (nCounter PanCancer Immune Profiling Panel Gene List.xlsx).

### Statistical analysis

Data analysis were performed using GraphPad Prism 7.02 and 8.02 (GraphPad Software Inc.). Outliers were identified by ROUT (*Q* = 1%) test and removed before statistical analyses were conducted. Information on data sets and statistical analysis for each experiment is reported in the main text and figure legends. *P* < 0.05 was considered statistically significant. Graphs showing percentage of animals with metastases also display the number of mice with metastases over the total number of mice in each treatment group. Graphs displaying single data points can be found in Supplementary Fig. 9.

### Reporting summary

Further information on research design is available in the Nature Research Reporting Summary linked to this article.

## Supplementary information

Supplementary Information

Reporting Summary

## Data Availability

The data that support the findings of this study are available from the corresponding author upon reasonable request. Data from the NanoString study have been deposited in NCBI’s Gene Expression Omnibus^56^ and are accessible through GEO Series accession number GSE174638.

## Code availability

All data analyses undertaken in R packages and functions contained therein, as described in the ‘Methods’ section.
