# Supplementary material for: IL-1B drives opposing responses in primary tumours and bone metastases; harnessing combination therapies to improve outcome in breast cancer
Source: NPJ Breast Cancer. 2021 Jul 21;7:95. doi: 10.1038/s41523-021-00305-w (PMC8295314; doi:10.1038/s41523-021-00305-w)
Supplement: Supplementary file 1 — Supplementary Information [file 41523_2021_305_MOESM1_ESM.pdf]

Supplementary Figures with Legends

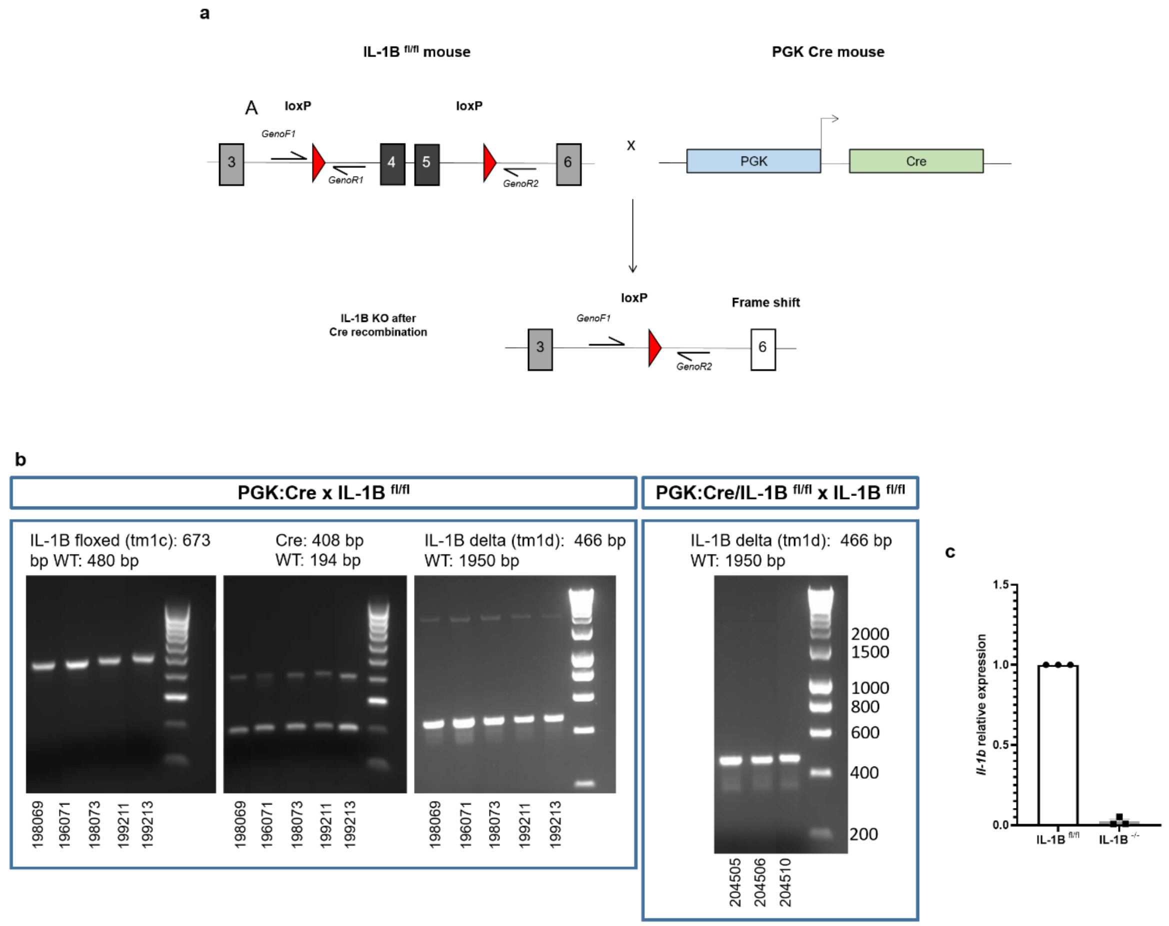

Supplementary Figure 1. Generation of IL-1B depleted mice. a) Diagram showing strategy to generate IL-1B ablated mice, generated by crossing mice in which exon 4-5 of the *Il-1b* gene is flanked with LoxP site (IL-1B$^{fl/fl}$) with mice expressing Cre recombinase under a ubiquitous phosphoglycerate kinase (PGK) promoter and then by backcrossing into IL-1B$^{fl/fl}$ mice. b) A representative genotyping PCR result for the identification of PGK:Cre x IL-1B$^{fl/fl}$ and IL-1B$^{-/-}$ mice, using primers (Geno F1/R1), (Cre) and (Geno F1/R2) (primer sequences in Methods section), is shown. c) Real-time PCR analysis from lungs isolated from IL-1B$^{fl/fl}$ and IL-1B$^{-/-}$ was performed to confirm IL-1B deletion. All gels derive from the same experiment and they were processed in parallel.

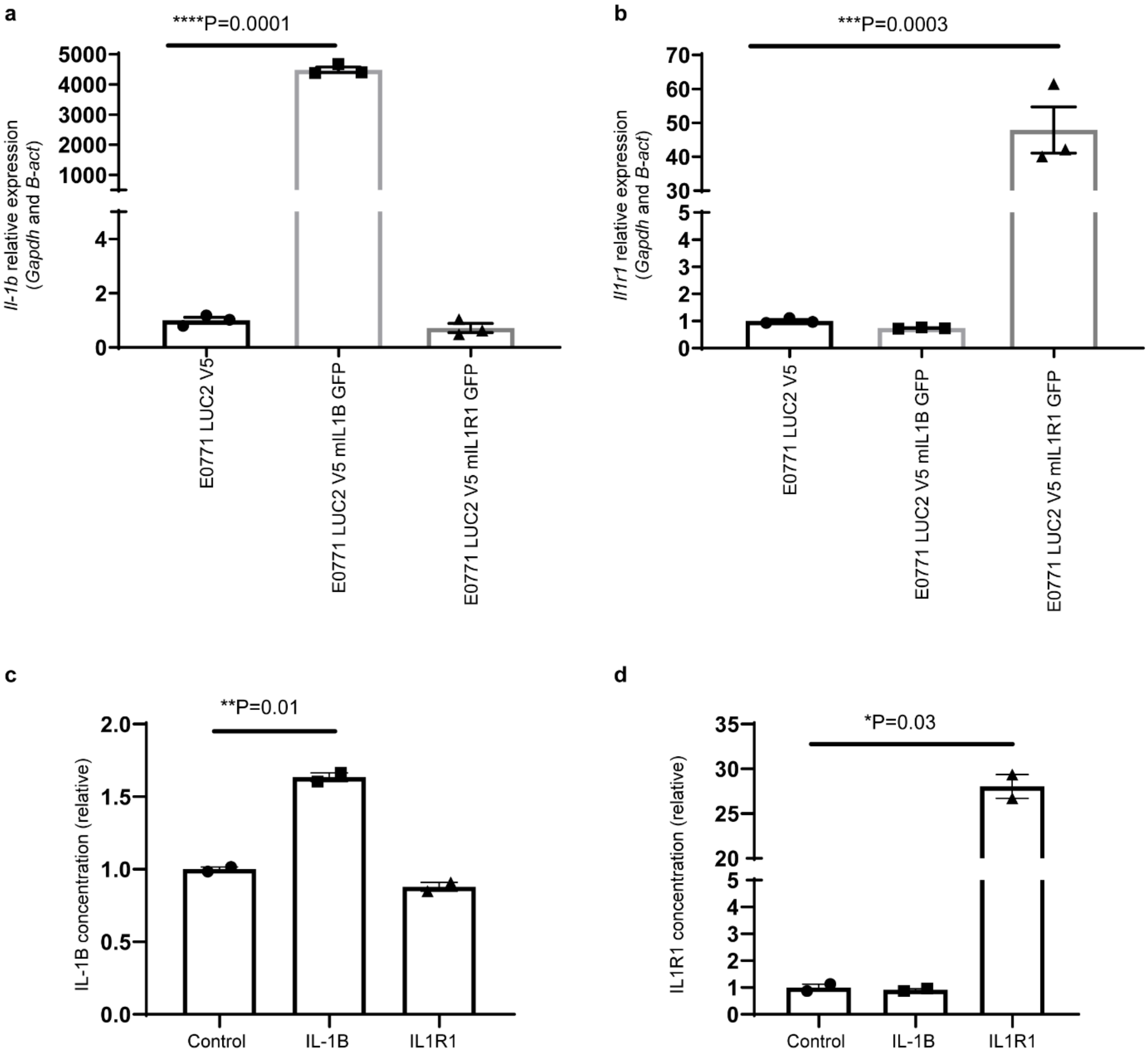

Supplementary Figure 2. Validation of IL-1B and IL-1R1 over-expression in E0771 cells. a) *Il-1b* relative expression in IL-1B over-expressing, IL1R1 over-expressing and control E0771 cell lines measured by Real-time PCR. b) *Il1r1* relative expression in IL-1B over-expressing, IL1R1 over-expressing and control E0771 cell lines measured by Real-time PCR. Data are shown as mean ± SEM, One-way ANOVA with Dunnet's post hoc test. c) Quantification of IL-1B protein levels in IL-1B over-expressing, IL1R1 over-expressing and control E0771 cell lines using ELISA. d) Quantification of IL1R1 protein levels in IL-1B over-expressing, IL1R1 over-expressing and control E0771 cell lines using ELISA. Data are mean ± SEM, Two-tailed Unpaired t-test with Welch's corrections.

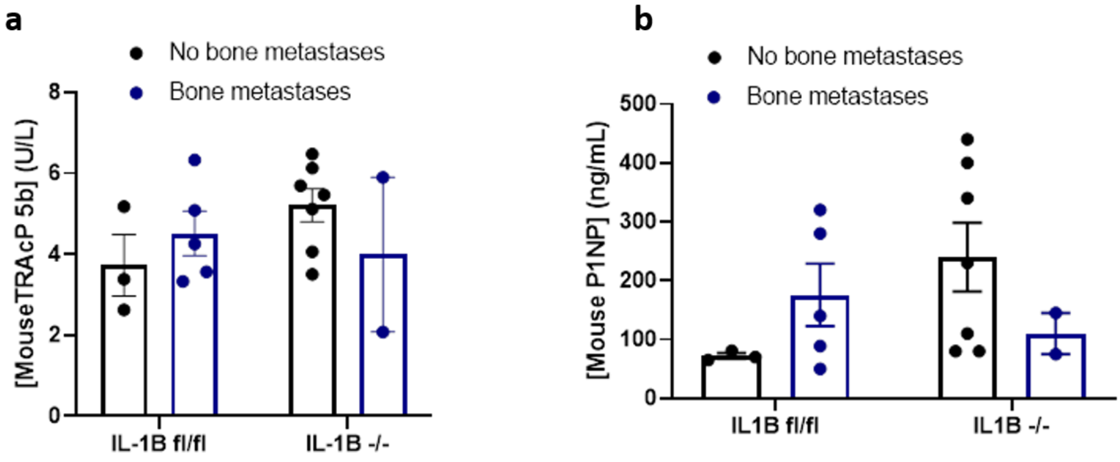

Supplementary Figure 3. IL-1B does not affect the activity of osteoclasts and osteoblasts in mice injected with tumour cells. a) Osteoclast and b) osteoblast activity (TRAcP and P1NP ELISA, respectively) in IL-1B$^{fl/fl}$ and IL-1B$^{-/-}$ mice injected with tumour cells. Samples were grouped in no bone metastases and bone metastases based on bioluminescence imaging performed *ex vivo*. Data are mean+/- SEM. Two-way ANOVA with Sidak's multiple comparison tests.

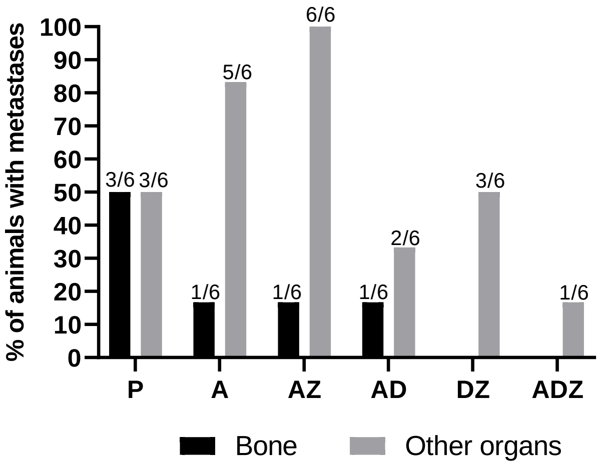

Supplementary Figure 4. Combining Anakinra with Dox+Zol is superior at inhibiting bone metastasis when compared to Anakinra+Zol and Anakinra+Dox. This graph shows the percentage of animals with metastases upon intra-cardiac (IC) injection of wild-type E0771 in C57BL/6J mice treated with Placebo (P), Anakinra (A), Anakinra+Zol (AZ), Anakinra+Dox (AD), Dox+Zol (DZ), Anakinra+Dox+Zol (ADZ). This graph shows the same dataset represented in Figure 6H, with the addition of the treatment groups Anakinra+Zol and Anakinra+Dox. The percentage of animals showing metastases is calculated based on n=6 mice in each treatment group and the number of animals showing metastasis is displayed on each bar.

A

GO: Biological Process	Adjusted P-value	Intersections
immune system process	2.83E-09	BLK,BST2,CAMP,CCR1,CCR7,CD14,IFITM2,IL21,IL25,IL6,ITGA2B,JAM3,LAMP3,MPO,SELL,TGFB2,THBS1,TREM1,VEGFC
immune response	1.31E-07	BLK,BST2,CAMP,CCR1,CCR7,CD14,IFITM2,IL21,IL25,IL6,JAM3,LAMP3,TGFB2,THBS1,TREM1
response to cytokine	4.58E-07	BST2,CAMP,CCR1,CCR7,CD14,CXCR4,IFITM2,IL13RA1,IL6,LAMP3,THBS1,VEGFC
cytokine production	9.55E-07	BST2,CAMP,CCR7,CD14,IL21,IL25,IL6,TGFB2,THBS1,TNFRSF9,TREM1
response to external stimulus	2.19124E-06	BST2,CAMP,CCR1,CCR7,CD14,CXCR4,IFITM2,IL21,IL25,IL6,JAM3,MPO,SELL,TGFB2,THBS1,TREM1,VEGFC
regulation of localization	2.65398E-06	BLK,BST2,CAMP,CCR1,CCR7,CD14,CXCR4,IL11,IL6,ITGA2B,JAM3,SELL,TGFB2,THBS1,TIE1,TNFRSF9,VEGFC
cell chemotaxis	3.21569E-06	CCR1,CCR7,JAM3,SELL,TGFB2,THBS1,TREM1,VEGFC
regulation of immune system process	4.41171E-06	BLK,BST2,CCR1,CCR7,CD14,IL21,IL6,ITGA2B,JAM3,SELL,TGFB2,THBS1,VEGFC
regulation of cell migration	5.82111E-06	BST2,CCR1,CCR7,CXCR4,ITGA2B,JAM3,SELL,TGFB2,THBS1,TIE1,VEGFC
myeloid leukocyte migration	7.03442E-06	CCR1,CCR7,JAM3,SELL,TGFB2,TREM1,VEGFC

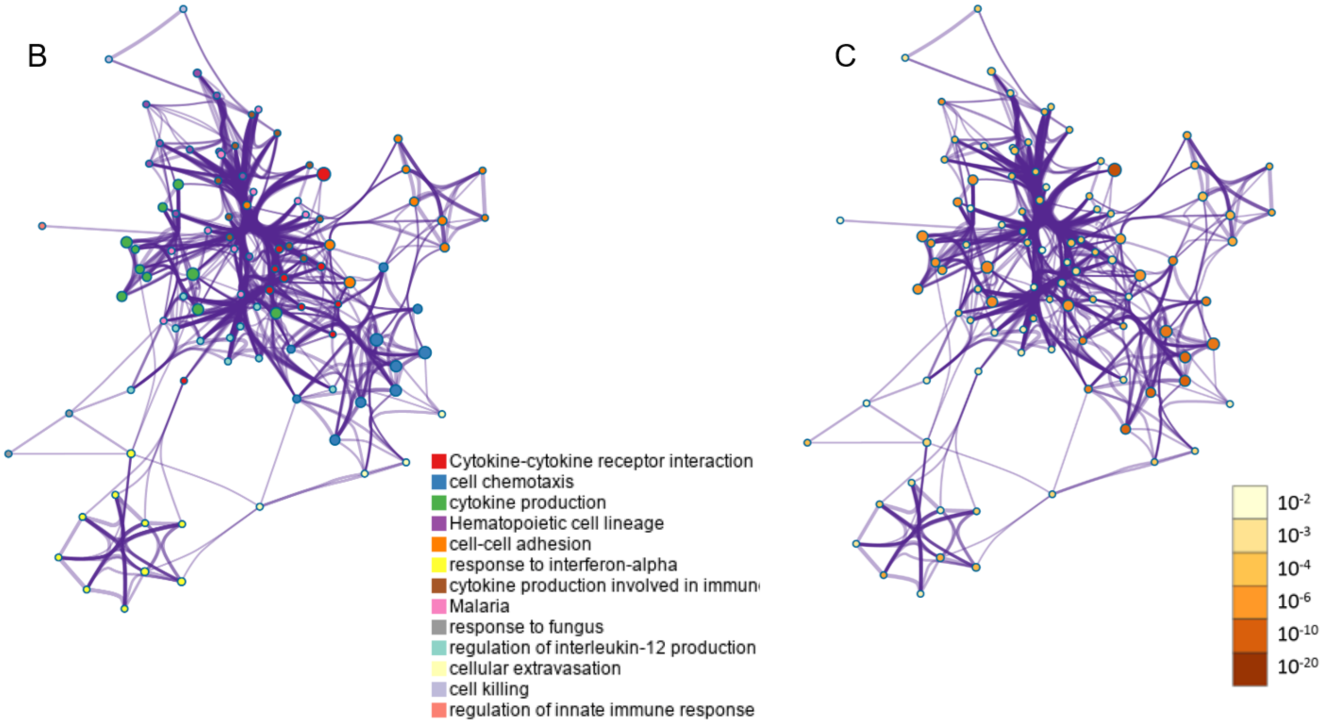

Supplementary Figure 5. GO analysis Anakinra vs Doz+Zol. a) The table displays the enrichment of the top10 immune-related GO terms (biological process) following treatment of tumour-bearing mice with A or DZ. b) Network of enriched terms colored by cluster ID. Nodes sharing the same cluster ID are closed to each other. c) Network of enriched terms colored by p-value. Terms containing more genes have a more significant p-value. Analysis in a) was performed using g: Profiler. Analysis in b) and c) was performed using Metascape.

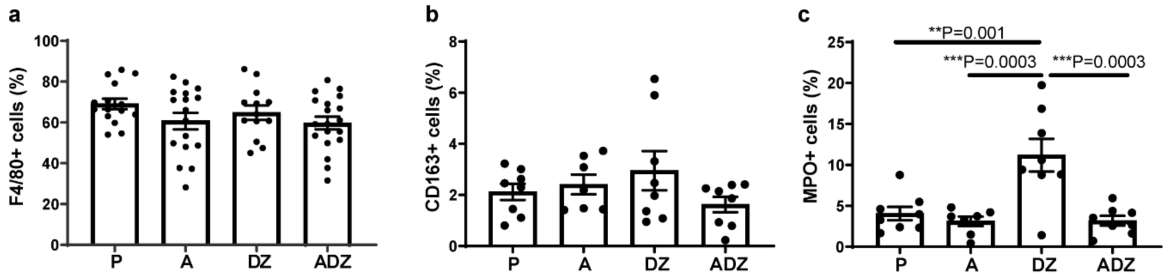

Supplementary Figure 6. Innate immune cell infiltration in primary tumours upon single and combination treatments. Immunohistochemistry for a) F4/80$^+$ macrophages, b) CD163$^+$ macrophages, c) MPO$^+$ neutrophils. Scoring of positive and negative cells is performed on one or more sections from each mammary tumour collected from mice treated with Placebo, Anakinra, Dox+Zol, Anakinra+Dox+Zol. Data are mean +/SEM. One-way ANOVA with Tukey's multiple comparisons test.

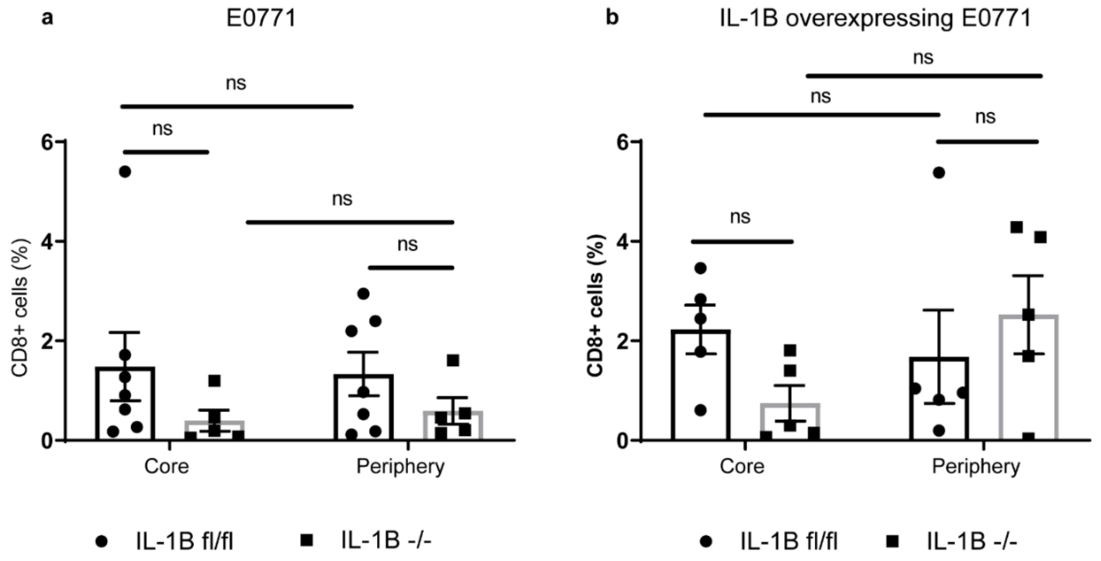

Supplementary Figure 7. CD8$^+$ T cells in primary tumours in IL-1B$^{fl/fl}$ and IL-1B$^{-/-}$ mice. CD8$^+$ T cell infiltration in the core and periphery of primary tumours originated from a) wild-type and b) IL-1B overexpressing E0771 cells injected in IL-1B$^{fl/fl}$ and IL-1B$^{-/-}$ mice. The number of T cells has been determined in whole tumour sections and percentages calculated over the number of negative cells. a) Core and periphery (IL-1B $^{fl/fl}$ n=7; IL-1B $^{-/-}$ n=5); b) Core and periphery (IL-1B $^{fl/fl}$ n=5; IL-1B $^{-/-}$ n=5) Data are mean +/- SEM. Two-way ANOVA with Sidak's multiple comparison tests.

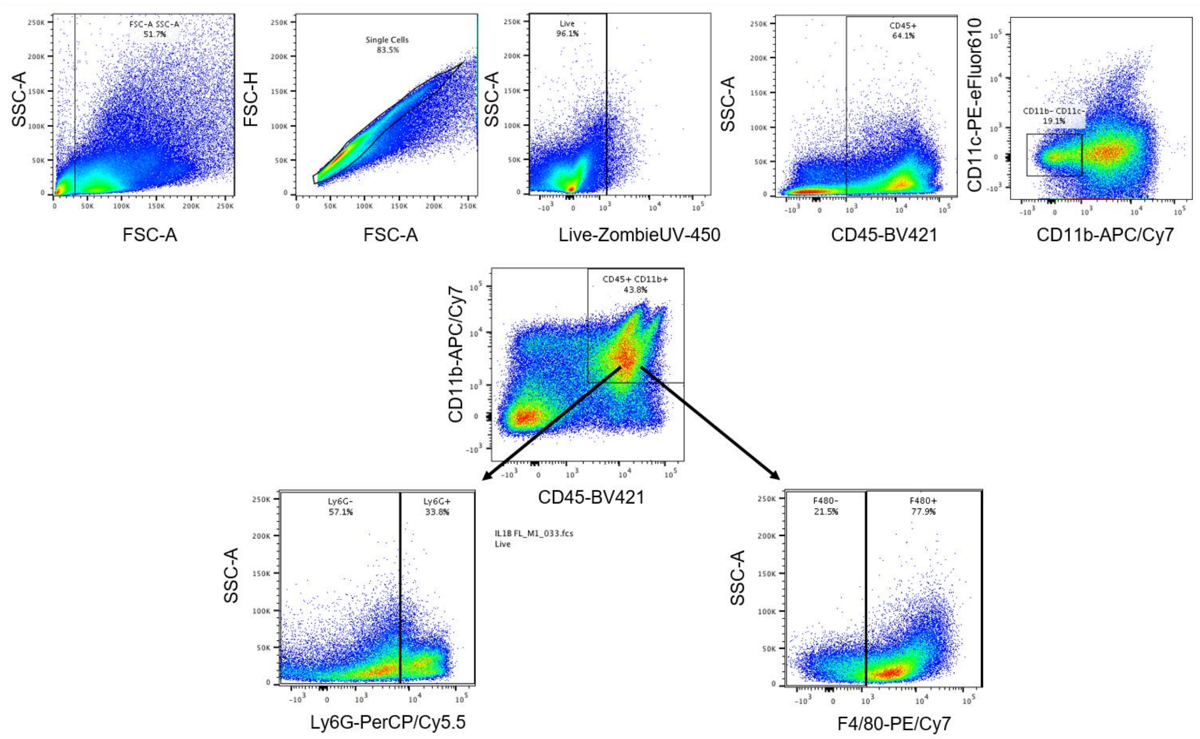

Supplementary Figure 8. Gating strategy. Representative dot plots illustrating the gating strategy for the identification of immune cell populations isolated from the bone marrow of tumour-bearing IL-1B$^{fl/fl}$ and IL-1B$^{-/-}$ mice.

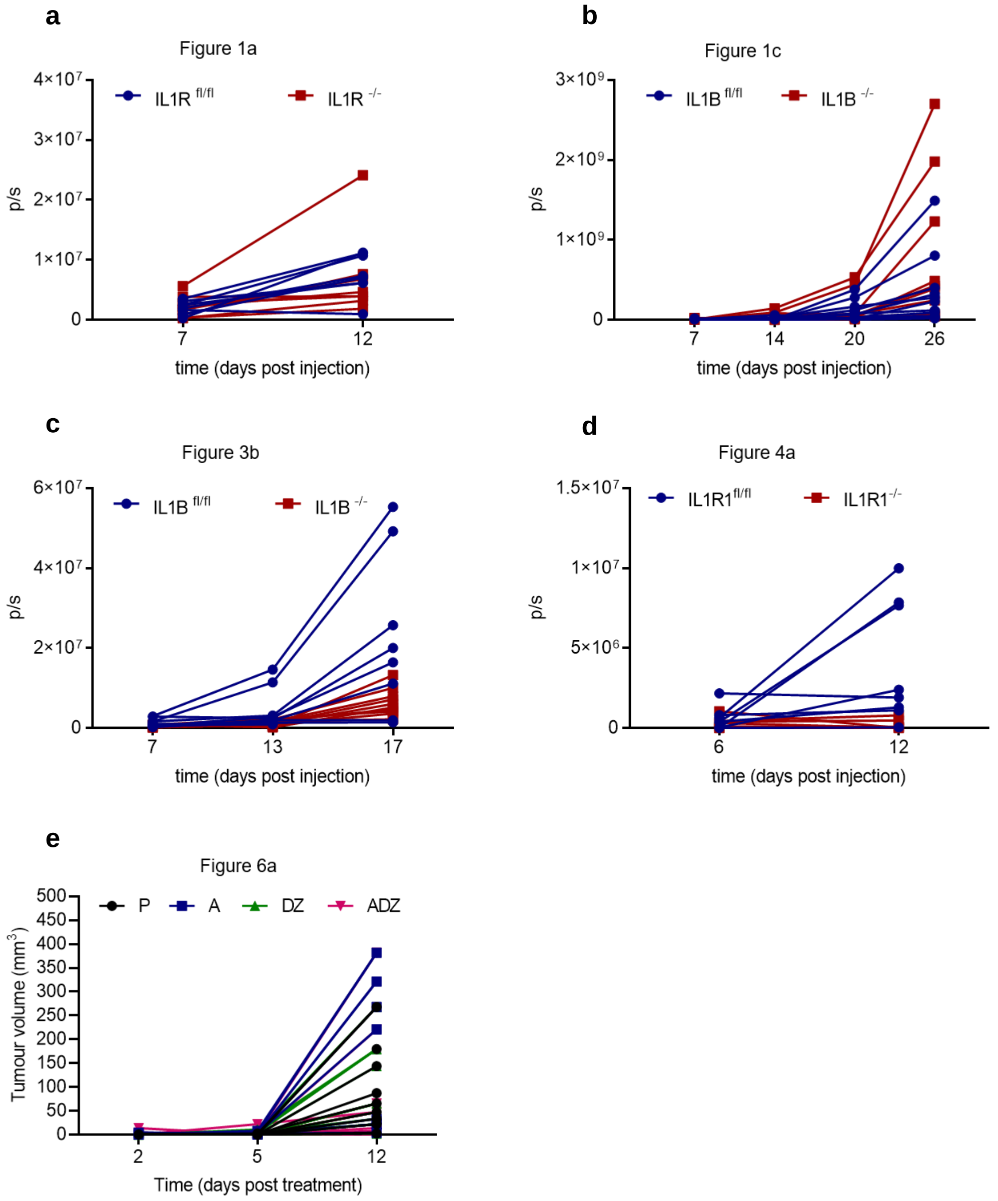

Supplementary Figure 9. Single data point graphs. Quantification of tumour growth using BLI. Graphs showing single data points are generated using the same datasets displayed in the main manuscript: a) Figure 1a; b) Figure 1c; c) Figure 3b; d) Figure 4a; e) Figure 6a

Supplementary Table 1

Primary antibody	Antigen retrieval	Dilution	Catalogue number	Secondary antibody	Dilution	Catalogue number
Rat anti-F4/80 (clone Cl: A3-1)	10µg/ml proteinase K	1:80	MCA497 (Serotec)	Biotinylated goat anti-rat	1:500	112-066-003, Jackson ImmunoResearch
Rabbit anti-iNOS	Citrate buffer	1:200	Ab15323, Abcam	Biotinylated anti-rabbit	1:500	111-066-003, Jackson ImmunoResearch
Rabbit anti-CD163 (clone EPR19518)	TRIS-EDTA (pH 9)	1:200	Ab182422, Abcam	Biotinylated anti-rabbit		
Rabbit anti-MPO	Citrate buffer	1:200	Ab9535, Abcam	Biotinylated anti-rabbit		
Rat-anti CD8a (clone 4SM15)	Citrate buffer	1:200	14-0808-80 (Invitrogen)	Biotinylated goat anti-rat	1:500	112-066-003, Jackson ImmunoResearch
Rabbit anti-Granzyme B	Citrate buffer	1:100	ab4059 Abcam	Biotinylated anti-rabbit	1:500	111-066-003, Jackson ImmunoResearch
Rat anti-CD34 (clone MEC14.7)	Citrate buffer	1:100	MCA1825 BioRad (Serotec)	Biotinylated goat anti-rat	1:500	112-066-003, Jackson ImmunoResearch
Mouse anti-PCNA (clone pc-10)	Citrate buffer	1:100	Santa-Cruz, cat no. sc56	Biotinylated anti-mouse IgG	1:250	M.O.M Kit (BMK-2202)
Rabbit anti-Cleaved Caspase 3	Citrate buffer	1:200	AF835 (VectorLabs)	Biotinylated anti-rabbit	1:500	111-066-003, Jackson ImmunoResearch

Supplementary Table 2

	**Fluorophore**	**Catalogue number (BioLegend)**	**Dilution**
Viability	Zombie UV	423107	1:100
CD45 (clone 30-F11)	BV421™	103134	1:100
CD11b (clone M1-70)	APC/Cy7™	101217	1:100
Ly6G (clone 1A8)	PerCP/Cy5.5™	127616	1:100
F4/80 (clone BM8)	PE/Cy7™	123114	1:100
CD11c (clone N418)	PE-eFluor610™	61-0114082	1:100
