# Supplementary material for: IL-1B drives opposing responses in primary tumours and bone metastases; harnessing combination therapies to improve outcome in breast cancer
Source: NPJ Breast Cancer. 2021 Jul 21;7:95. doi: 10.1038/s41523-021-00305-w (PMC8295314; doi:10.1038/s41523-021-00305-w)
Supplement: Supplementary file 2 — Reporting Summary [file 41523_2021_305_MOESM2_ESM.pdf]

nature research

Corresponding author(s): Penelope Ottewell

Last updated by author(s): Jun 29, 2021

Reporting Summary

Nature Research wishes to improve the reproducibility of the work that we publish. This form provides structure for consistency and transparency in reporting. For further information on Nature Research policies, see our Editorial Policies and the Editorial Policy Checklist.

Statistics

For all statistical analyses, confirm that the following items are present in the figure legend, table legend, main text, or Methods section.

n/a	Confirmed	
☐	☒	The exact sample size (*n*) for each experimental group/condition, given as a discrete number and unit of measurement
☐	☒	A statement on whether measurements were taken from distinct samples or whether the same sample was measured repeatedly
☐	☒	The statistical test(s) used AND whether they are one- or two-sided<br>*Only common tests should be described solely by name; describe more complex techniques in the Methods section.*
☐	☒	A description of all covariates tested
☐	☒	A description of any assumptions or corrections, such as tests of normality and adjustment for multiple comparisons
☐	☒	A full description of the statistical parameters including central tendency (e.g. means) or other basic estimates (e.g. regression coefficient) AND variation (e.g. standard deviation) or associated estimates of uncertainty (e.g. confidence intervals)
☐	☒	For null hypothesis testing, the test statistic (e.g. *F*, *t*, *r*) with confidence intervals, effect sizes, degrees of freedom and *P* value noted<br>*Give P values as exact values whenever suitable.*
☒	☐	For Bayesian analysis, information on the choice of priors and Markov chain Monte Carlo settings
☒	☐	For hierarchical and complex designs, identification of the appropriate level for tests and full reporting of outcomes
☒	☐	Estimates of effect sizes (e.g. Cohen's *d*, Pearson's *r*), indicating how they were calculated

Our web collection on statistics for biologists contains articles on many of the points above.

Software and code

Policy information about availability of computer code

Data collection	Bioluminescence in vivo imaging was performed using IVIS Lumina II in Vivo Imaging System (PerkinElmer) and Living Image® 4.5.4 software<br>FACS DIVA software was used to collect flow cytometry data, acquired by using a BD LSRII™ flow cytometer (Flow Cytometry Core Service, University of Sheffield)<br>Real-time PCR was performed using 7900HT PCR system (Applied Biosystems) (Genomic Core Facility, University of Sheffield)<br>ELISAs and Cell viability (MTT cell proliferation assay) were assessed using EnSight multimode Plate reader (PerkinElmer).<br>µCT analysis was carried out using a Skyscan 1172 X-ray–computed µCT scanner (Skyscan) equipped with an X-ray tube (voltage, 49 kV; current, 200 mA) and a 0.5-mm aluminum filter.<br>Tissue sections were digitally scanned using Panoramic 250 Flash III slide scanner (3DHISTECH).<br>Cell migration images were acquired using InvitrogenTM EVOS TM FL auto imaging system (Thermo Fisher Scientific).
Data analysis	Living Image® 4.5.4 software was used to analyse bioluminescence in vivo images<br>FlowJo v10 was used to analyse flow cytometry data and perform gating startegy<br>Data analysis were performed using GraphPad Prism 7.02 and 8.02 (GraphPad Software Inc.).<br>µCT image analysis was carried out using Skyscan NRecon Reconstruction software and CTVox<br>Nanostring analysis was performed using nSolverTM 4.0 and Advanced Analysis 2.0 (NanoString Technologies) and R.<br>Quantification of percentage of positive cells on tissue sections after Immunohistochemistry was performed using QuPath: Open source software for digital pathology image analysis.<br>Cell migration images were analysed using ImageJ

For manuscripts utilizing custom algorithms or software that are central to the research but not yet described in published literature, software must be made available to editors and reviewers. We strongly encourage code deposition in a community repository (e.g. GitHub). See the Nature Research guidelines for submitting code & software for further information.

Data

Policy information about availability of data

All manuscripts must include a data availability statement. This statement should provide the following information, where applicable:

- Accession codes, unique identifiers, or web links for publicly available datasets
- A list of figures that have associated raw data
- A description of any restrictions on data availability

The data that support the findings of this study are available from the corresponding author upon reasonable request. Data from the NanoString study have been deposited in NCBI's Gene Expression Omnibus and are accessible through GEO Series accession number GSE174638.

Field-specific reporting

Please select the one below that is the best fit for your research. If you are not sure, read the appropriate sections before making your selection.

☒ Life sciences ☐ Behavioural & social sciences ☐ Ecological, evolutionary & environmental sciences

For a reference copy of the document with all sections, see nature.com/documents/nr-reporting-summary-flat.pdf

Life sciences study design

All studies must disclose on these points even when the disclosure is negative.

Sample size	For in vivo experiments, power calculations were carried on based on previous experience with mouse models of intra-ductal and intra-cardiac tumour cell injections (Tulotta et al, Clinical Cancer Res 2019; Eyre et al., Nat Commun 2019; Lefley et al., Breast Cancer Res 2019)
Data exclusions	Outliers were identified by ROUT (Q=1%) test in GraphPad and removed before statistical analyses were conducted. This exclusion criteria was pre-determined.
Replication	In vivo experiments conducted in IL-1B KO, IL1R1 KO and relative controls: considering the variability in the litter size and in order to reach the recommended sample size, tumour cell injection was performed on multiple days (with the same cell suspension been injected in both KO and fl/fl on the same day). The combination therapy was performed using 2 different mouse models of breast cancer and 2 different tumour cell lines.
Randomization	For intervention studies, animals were injected with tumour cells, imaged and randomised in groups prior to the start of the pharmacological treatment.
Blinding	Scoring of tissue sections after immunohistochemistry was performed by using an automated analysis method, by setting a signal threshold in Qpath to be used in every section of the same experiment.

Reporting for specific materials, systems and methods

We require information from authors about some types of materials, experimental systems and methods used in many studies. Here, indicate whether each material, system or method listed is relevant to your study. If you are not sure if a list item applies to your research, read the appropriate section before selecting a response.

Materials & experimental systems

n/a	Involved in the study	
☐	☒	Antibodies
☐	☒	Eukaryotic cell lines
☒	☐	Palaeontology and archaeology
☐	☒	Animals and other organisms
☒	☐	Human research participants
☒	☐	Clinical data
☒	☐	Dual use research of concern

Methods

n/a	Involved in the study	
☒	☐	ChIP-seq
☐	☒	Flow cytometry
☒	☐	MRI-based neuroimaging

Antibodies

Antibodies used	Antibodies used for Immunohistochemistry: Rat anti-F4/80 (clone Cl: A3-1) MCA497 (Serotec); Rabbit anti-iNOS Ab15323, Abcam; Rabbit anti-CD163 (clone EPR19518) Ab182422, Abcam; Rabbit anti-MPO Ab9535, Abcam; Rat-anti CD8a (clone 4SM15) 14-0808-80 (Invitrogen); Rabbit anti-Granzyme B ab4059 Abcam; Rat anti-CD34 (clone MEC14.7) MCA1825 BioRad (Serotec); Mouse anti-PCNA (clone pc-10) Santa-Cruz, cat no. sc56; Rabbit anti-Cleaved Caspase 3 AF835 (VectorLabs); Biotinylated goat anti-rat 112-066-003, Jackson ImmunoResearch; Biotinylated anti-rabbit 111-066-003, Jackson ImmunoResearch; Biotinylated anti-mouse IgG M.O.M Kit (BMK-2202).

Antibodies for flow cytometry (BioLegend): Viability Zombie UV (Catalogue number: 423107); CD45 (clone 30-F11) BV421™ (Catalogue number: 103134); CD11b (clone M1-70) APC/Cy7™ (Catalogue number: 101217); Ly6G (clone 1A8) PerCP/Cy5.5™ (Catalogue number: 127616); F4/80 (clone BM8) PE/Cy7™ (Catalogue number: 123114); CD11c (clone N418) PE-eFluor610™ Catalogue number: 61-0114082).

Validation	Antibodies for flow cytometry were validated for target species (mouse) using FMOs, single stains and compensation beads. Staining specificity in immunohistochemistry was tested on tissue sections by comparison with a negative control (only secondary antibody). Tissue sections were used as a positive control, when available. All antibodies were pre-validated by each supplier.

Eukaryotic cell lines

Policy information about cell lines

Cell line source(s)	As described by ATCC, The E0771 mammary tumor was first reported in 1948 as a spontaneous mammary carcinoma arising in a C57Bl/6 mouse (https://www.lgcstandards-atcc.org/products/all/CRL-3461.aspx#generalinformation). E0771 luc2 GFP were kindly provided by Jessalyn Ubellacker, from Professor Sandra McAllister's laboratory (Department of Medicine, Harvard Medical School, Boston, MA, USA). The E0771 cells used in the current study have been selected for their bone homing capabilities following two rounds of intra-cardiac tumour cell injection, in which bone metastatic clones have been isolated and re-injected. 4T1 luc2 were kindly provided by Ryan Bishop (Department of Oncology and Metabolism, University of Sheffield, UK). E0771 Luc2 V5 GFP, E0771 Luc2 V5 IL-1B GFP and E0771 Luc2 V5 IL1R1 cell lines were generated using lentiviral transduction as described in the Materials and Methods section of the manuscript.
Authentication	Cell lines were not authenticated. E0771 luc2 v5 GFP, IL-1B GFP and IL1R1 GFP were all made from the same parental line.
Mycoplasma contamination	Cells were routinely checked for mycoplasma infection, using a EZ PCR™ Mycoplasma Detection Kit (Biological Industries).
Commonly misidentified lines (See ICLAC register)	No misidentified cell lines were used in this study.

Animals and other organisms

Policy information about studies involving animals; ARRIVE guidelines recommended for reporting animal research

Laboratory animals	In vivo experiments were performed using 6-8-week-old female IL-1Bfl/fl (control) or IL-1B -/- mice (C57BL/6J background), IL1R1fl/fl (control), IL1R1-/- mice (C57BL/6J background), C57BL/6J and BALB/c. Ubiquitous knockout for IL-1B was obtained by backcrossing PGK;Cre; IL-1Bfl/fl to IL-1Bfl/fl mice.
Wild animals	The study did not involve wild animals
Field-collected samples	The study did not involve samples collected from the field
Ethics oversight	In vivo procedures were conducted in accordance with local guidelines and with UK Home Office approval under Project License (PPL) 70/8964 or P99922A2E, University of Sheffield, UK.

Note that full information on the approval of the study protocol must also be provided in the manuscript.

Flow Cytometry

Plots

Confirm that:

☒ The axis labels state the marker and fluorochrome used (e.g. CD4-FITC).

☒ The axis scales are clearly visible. Include numbers along axes only for bottom left plot of group (a 'group' is an analysis of identical markers).

☒ All plots are contour plots with outliers or pseudocolor plots.

☒ A numerical value for number of cells or percentage (with statistics) is provided.

Methodology

Sample preparation	Cryopreserved bone marrow was defrosted and washed in ice-cold PBS supplemented with 1% v/v FBS (FACS buffer). Samples were aliquoted and incubated with fluorochrome-conjugated antibodies and live/dead dye (Supplementary Table 2) (diluted 1:100) for 45 min on ice. After washing in FACS buffer, samples were resuspended in 500 µl FACS buffer.
Instrument	Experiments were performed using a BD LSRII™ flow cytometer (FACS Diva software)
Software	Analysis and gating strategy were performed using FlowJo software v10 (FlowJo LLC)
Cell population abundance	No cell sort was performed
Gating strategy	The FSC-A/SSC-A gate was used to included all cells and exclude debris based on cell morphology. Doublets (FSC-H/FSC-A)

Gating strategy

and dead cells (SSC-A/ZombieUV-450) were excluded. Immune cells were then gated based on their specific markers, as shown in the Gating Strategy in Supplementary Figure 7.

☒ Tick this box to confirm that a figure exemplifying the gating strategy is provided in the Supplementary Information.
